# Development and diagnostic performance of a sensitive multiplex pneumonia pathogen identification assay for emergency department patients with pneumonia

**DOI:** 10.1128/spectrum.00811-25

**Published:** 2025-09-03

**Authors:** Pei-Yi Tsui, Chia-Wei Hong, Yi-Da Tsai, Chu-Yang Chien, Yi-Ling Chen, Hsin-Hsien Huang, Chin-Mao Hung, Feng-Ping Lin, Chung-Chih Liang, Chih-Yuan Lin, Shih‑Hung Tsai, Hui-Ling Hsu

**Affiliations:** 1 Graduate Institute of Medical Sciences, National Defense Medical University 71548, Taipei, Taiwan, Republic of China; 2 Institute of Preventive Medicine, National Defense Medical University 71548, New Taipei City, Taiwan, Republic of China; 3 Department of Emergency Medicine, Tri-Service General Hospital, National Defense Medical University 71548, Taipei, Taiwan, Republic of China; 4 Department of Materials Science and Engineering, National Taiwan University of Science and Technology, Taipe, Taiwan, Republic of China; 5 Division of Cardiovascular Surgery, Department of Surgery, Tri-Service General Hospital, National Defense Medical University 71548, Taipei, Taiwan, Republic of China; 6 National Defense Medical University 71548, Taipei, Taiwan, Republic of China; 7 Taichung Armed Forces General Hospital 38003, Taichung, Taiwan, Republic of China; 8 Department of Physiology and Biophysics, Graduate Institute of Physiology, National Defense Medical University 71548, Taipei, Taiwan, Republic of China; 9 Department of Microbiology and Immunology, National Defense Medical University 71548, Taipei, Taiwan, Republic of China; Indiana University School of Medicine, Indianapolis, Indiana, USA

**Keywords:** multiplex, pathogen detection, pneumonia, emergency departments

## Abstract

Pneumonia imposes a significant global health burden, with high morbidity and mortality, and challenges in pathogen identification due to diverse etiologies. Conventional culture-based methods are time-consuming and insufficient for fastidious organisms, necessitating advanced diagnostic tools for rapid and accurate pathogen identification, especially in emergency department (ED) settings. To address this, we developed the pneumonia pathogen identification (PPID) assay, a multiplex diagnostic platform based on the single-stranded multiplex PCR amplicons with suspension bead array technology. The PPID assay detects 27 bacterial and viral pathogens within 6 hours. Analytical validation using nucleic acids from reference strains or synthetic oligonucleotides demonstrated high specificity and sensitivity (5–500 copies per reaction). In 135 ED cases with pneumonia, the PPID assay significantly outperformed microbiological culture methods (77.8% vs 45.2%), increasing the overall detection rate to 85.9% when combined. It effectively identified polymicrobial infections in 48.6% of positive cases, including bacterial-viral co-infections, with *Klebsiella pneumoniae* being the most frequently identified pathogen overall and in mixed infections. These results highlight the PPID assay’s rapid and comprehensive diagnostic capability, facilitating early and targeted treatment, particularly in acute care settings. This innovative approach addresses critical unmet clinical needs and improves patient outcomes.

**IMPORTANCE:**

The pneumonia pathogen identification assay is a high-throughput multiplex molecular test that detects 27 respiratory pathogens within 6 hours for up to 96 samples. It exhibits high specificity and sensitivity and enhances detection of fastidious pathogens and co-infections, addressing critical diagnostic challenges in emergency settings.

## INTRODUCTION

Pneumonia is one of the most common infections encountered in emergency departments (EDs), associated with high rates of morbidity and mortality (1–4). Due to its rapid transmission, pneumonia can lead to localized outbreaks or even global pandemics, as evidenced by the severe acute respiratory syndrome (SARS) and the coronavirus disease 2019 (COVID-19) pandemics. Pneumonia is classified by the location of acquisition into community-acquired pneumonia (CAP), hospital-acquired pneumonia (HAP), and healthcare-associated pneumonia (HCAP) (4–8). It is also categorized by etiology, such as bacterial, viral, or fungal pneumonia.

Accurate pathogen identification in clinical settings is ideal for guiding appropriate treatment and improving patient outcomes. However, the presence of diverse causative pathogens, along with similar clinical manifestations—such as fever, cough, dyspnea, and chest discomfort—makes it challenging to distinguish between etiologies based on symptoms alone. In the ED, clinicians often need to initiate empirical treatment within hours of a patient’s presentation, typically before pathogen identification is available. This time-sensitive nature of emergency care, combined with diagnostic uncertainty, contributes to the overuse of broad-spectrum antibiotics and increases the risk of inappropriate therapy. Furthermore, patients may present with co-infections, atypical infections, or a history of prior antibiotic exposure, which further complicates the diagnostic process. These challenges underscore the urgent need for rapid, comprehensive, and accurate diagnostic tools.

Conventional microbiological culture (MC) methods remain the gold standards for pneumonia pathogen detection. However, MC methods come with the inconveniences such as prolonged turnaround time and low sensitivity, which reduce their effectiveness. In addition, MC methods are not particularly suitable for detecting pathogens suppressed by prior antibiotic use, as well as for fastidious or atypical bacteria that grow slowly or require specialized culture conditions. Furthermore, routine viral cultures are not typically performed in clinical settings due to their complexity, extended turnaround time, and the need for specialized laboratory facilities. Added together, these limitations of conventional culture-based techniques often fall short of ED demands.

To address the limitations of traditional culture-based diagnostics, various molecular techniques have been developed, including PCR, real-time PCR, microarray, FilmArray, and next-generation sequencing (NGS), offering rapid and sensitive detection (9–14). However, these methods also have certain limitations, such as a restricted pathogen coverage (15, 16) or extended processing times, as with NGS, which may take several days (17–19).

Hsu *et al*. (20) developed the single-stranded multiplex PCR amplicons with suspension bead array (SSMP-SBA) assay, a high-throughput technology for multiplex pathogen identification. This technology utilizes a two-step multiplex PCR amplification and magnetic microsphere-based detection, achieving high specificity and sensitivity with a detection limit of 5–100 genome equivalents. It enables the simultaneous detection of 11 pathogens, targeting 15 genetic markers within 6 hours, multiplexing to identify two to six targets per sample with 100% accuracy, and is therefore particularly beneficial for detecting low-load pathogens and identifying co-infections, which are critical in managing acute infectious diseases.

To address the need for rapid and comprehensive pneumonia pathogen detection, we developed the pneumonia pathogen identification (PPID) assay, a multiplex diagnostic platform targeting 27 bacterial and viral pathogens, including common respiratory pathogens and fastidious or atypical bacteria such as *Mycobacterium tuberculosis*, *Bordetella pertussis*, *Legionella pneumophila*, *Mycoplasma pneumoniae*, and *Chlamydia pneumoniae*. The inclusion of *M. tuberculosis* is particularly critical due to its significant impact on global public health. Additionally, the assay encompasses etiological agents of viral pneumonia, such as influenza viruses, SARS-CoV-2, respiratory syncytial virus (RSV), and other viruses, totaling 10 viral targets. In this study, we evaluated its analytical performance and assessed its clinical utility in pneumonia cases presenting to EDs.

## MATERIALS AND METHODS

### Primer and probe design

For each target pathogen, specific primers and probes were designed based on pathogen sequences from GenBank (National Center for Biotechnology Information [NCBI], Bethesda, MD, USA) using DNASTAR Lasergene Software v.7.0 (DNASTAR Inc., Madison, WI, USA) and PrimerPlex Software v.1.5 (Premier Biosoft, Palo Alto, CA, USA). Among these, the Hin primer/probe set was designed to detect all serotypes and non-typeable strains of *Haemophilus influenzae*, while the Hib primer/probe set was designed exclusively for *H. influenzae* serotype b. The melting temperature values of primers and probes were set at 72°C–75°C and 50°C–55°C, respectively. Sequence specificity was verified using BLAST (NCBI). Unique forward/reverse (UF/UR) sequences were incorporated at the 5ʹ ends of pathogen-specific primers to enable second-stage PCR amplification using unique primers. The UR primers were 5ʹ-biotin-labeled and contained four phosphorothioate linkages at the five terminal bases. Probes were modified with a 5ʹ amino group and a 12-carbon spacer for efficient coupling to Bio-Plex Pro magnetic COOH beads (Bio-Rad, Hercules, CA, USA) via the carbodiimide coupling method, according to the manufacturer’s instructions. All primers and probes were synthesized by Integrated DNA Technologies (IDT), Inc. (Singapore). The details of pathogens, target genes, and sequences are listed in Table S1.

### Reference strains and controls

Positive controls for the validation of the analytical specificity and sensitivity, listed in Table S2, included nucleic acids from 14 bacterial reference strains and synthetic oligonucleotides targeting *M. tuberculosis*, *C. pneumoniae*, *M. pneumoniae,* and 10 respiratory viruses, due to the lack of available reference strains for these organisms. The reference strains were sourced from the Biomaterial Repository of the Institute of Preventive Medicine, National Defense Medical Center, Taiwan. The synthetic oligonucleotides were synthesized by IDT. Negative controls comprised 10 mM Tris buffer as a non-template control and oropharyngeal specimens from healthy volunteers as negative specimens.

### Patients and clinical specimens

Adult patients (≥20 years old) admitted to EDs of the Tri-Service General Hospital (TSGH) between September 2018 and September 2023 were eligible for inclusion. The inclusion criteria were (1) a clinical or radiographic diagnosis of acute lower respiratory tract infection or pneumonia, defined as the presence of at least two of the following symptoms: fever (>38°C), cough, sore throat, purulent sputum production, dyspnea, tachypnea, wheezing or stridor, hypoxemia, cyanosis, or respiratory distress, and (2) the need for intubation and mechanical ventilation. Exclusion criteria included the absence of clinical evidence of infection, insufficient clinical information, missing experimental data, or positive pathogen culture reports within the preceding week. Endotracheal aspirates (ETAs) were collected from all enrolled patients for pathogen detection and identification. A portion of each ETA specimen was submitted to the Department of Laboratory Medicine of the TSGH for conventional microbiological culture-based identification during patient treatment, which predates the PPID assay. The culture reports, but not the culture plates, were made available to us. The remaining aliquots were stored at −20°C and subsequently made available to us to be tested with the PPID assay in batches.

### Conventional MC methods

ETA specimens were routinely inoculated onto Columbia colistin-nalidixic acid agar, MacConkey agar, and chocolate agar plates, and incubated at 35°C–37°C for 24–48 hours. Bacterial identification was performed based on colony morphology, Gram’s staining, biochemical testing, or matrix-assisted laser desorption ionization time-of-flight mass spectrometry, as appropriate. The culture for fastidious or atypical bacteria (*M. tuberculosis, C. pneumoniae*, *L. pneumophila*, *M. pneumoniae,* and *B. pertussis*) and viruses was not conducted.

### Pretreatment of reference strains and respiratory specimens before nucleic acid extraction

For the reference strains, bacterial colonies from overnight culture plates were used to prepare a McFarland 3 suspension in phosphate-buffered saline, corresponding to an approximate bacterial density of 9.0 × 10⁸ colony-forming units (CFU)/mL (21), to minimize the differences in the concentrations of the various reference strains. ETA specimens were rinsed twice with sterile normal saline and liquefied using 7.5% Remel Sputasol (Thermo Fisher Scientific Inc., Waltham, MA, USA). A total of 500 µL of bacterial suspension, oropharyngeal specimens, or washed ETA specimens was homogenized and lysed via bead-beating with the Precellys Lysing Kit CK-01 and Precellys Evolution homogenizer (Bertin Technologies, Paris, France) following the manufacturer’s instructions. The processed lysate was centrifuged at 10,000 × *g* for 2 min, and 200 µL of the supernatant was subjected to nucleic acid extraction.

### Nucleic acid extraction

Total nucleic acids were extracted from a starting volume of 200 µL using the QIAamp DNA Mini Kit on a QIAcube Connect system (QIAGEN, Hilden, Germany) and eluted in 100 µL of elution buffer. DNA from the reference strains was quantified using the Qubit dsDNA HS Assay Kit on a Qubit 4.0 Fluorometer (Thermo Fisher Scientific Inc., Waltham, MA, USA).

### PPID assay

The PPID assay detects up to 17 bacterial and 10 viral pathogens in three steps: multiplex PCR, T7 exonuclease hydrolysis, and suspension bead array (SBA).

For virus detection, nucleic acids (RNA) were reverse-transcribed to cDNA using the SuperScript VILO cDNA Synthesis Kit (Invitrogen, Thermo Fisher Scientific, Waltham, MA, USA) prior to multiplex PCR. PCR amplification, T7 exonuclease hydrolysis, and hybridization reaction of SBA were performed on the Applied Biosystems ProFlex 96-well PCR System (Thermo Fisher Scientific, Waltham, MA, USA).

#### Multiplex PCR

Multiplex PCR involves two stages of PCR reactions. The first stage involves amplification using pathogen-specific primers with 5ʹ unique sequences, followed by a second-stage amplification with unique primers. Each 25 µL multiplex PCR reaction contained 4 µL of template DNA/cDNA, 80 nM of each pathogen-specific primer, 1 µM of UF/UR primers, 16 nM RNase P (RP) primer pairs, 2.5 mM MgCl_2_, 200 µM of each deoxyribonucleotide triphosphate, 1× AmpliTaq Gold Buffer, and 0.625 units of AmpliTaq Gold DNA Polymerase (Applied Biosystems, Foster City, CA, USA). Thermal cycling conditions included initial activation at 95°C for 10 min; first-stage PCR, 25 cycles of 95°C for 30 s, 65°C for 60 s, and 72°C for 30 s; second-stage PCR, 35 cycles of 95°C for 30 s, 50°C for 30 s, and 72°C for 30 s, with a final extension at 72°C for 7 min.

#### T7 exonuclease hydrolysis

Multiplex PCR products were hydrolyzed with T7 exonuclease to yield single-stranded products as described previously (20). Briefly, 18 µL of multiplex PCR products was mixed with 21 units of T7 exonuclease (New England Biolabs, Ipswich, MA, USA) in a 36 µL reaction and incubated at 25°C for 40 min.

#### Suspension bead array

Seventeen microliters of single-stranded products and 2,500 probe-coupled magnetic beads from each probe set in 33 µL of the hybridization buffer (3M tetramethylammonium chloride, 0.1% sarkosyl, 50 mM Tris-HCl, and 4 mM EDTA) were mixed and incubated at 95°C for 10 min and 50°C for 30 min for hybridization. After three times of washing with 100 µL of hybridization buffer on the BioTek ELx50 microplate washer (Agilent Technologies, Santa Clara, CA, USA), the magnetic beads were mixed with 2.5 µg/mL of BD Pharmingen R-Phycoerythrin (PE) Streptavidin (BD Biosciences, San Jose, CA, USA), incubated at room temperature for 5 min with shaking, washed three times with hybridization buffer, and finally suspended in 100 µL of hybridization buffer. The PE-labeled beads were analyzed on the Bio-Plex 200 System (Bio-Rad Laboratories, Hercules, CA, USA) using Bio-Plex Manager software v.5.0 (Bio-Rad Laboratories, Hercules, CA, USA) with the High RP1 setting. At least 50 beads were analyzed for each probe set, and the results were reported as median fluorescence intensity (MFI).

### Data analysis and interpretation

The results for the probe sets were converted from MFIs into signal-to-noise ratios (SNRs), calculated as the ratio of the sample’s MFIs (“Signals”) to those of the negative specimens (“Noises”). The positive cutoff value for each target (as shown in Table 1 and 2) was defined as the mean SNR of negative specimens and non-target pathogens tested with the PPID assay, plus three standard deviations. For each probe set, a result is interpreted as positive if the SNR values of all replicates exceed the positive cutoff value; otherwise, it is reported as “not detected” (ND).

**TABLE 1:** Analytical specificity of the PPID assay for bacterial targets^*a*^

	Probe set																
Template	Aba	Bpe	Cpn	Eco	Hin	Hib	Kpn	Lpn	Mca	Mpn	Mtb	Nme	Pae	Sau	Sma	Spn	Spy
*Acinetobacter baumannii*	***315.3***	1.4	0.7	0.6	1.0	1.2	0.1	0.9	1.4	1.1	1.3	1.5	1.0	0.7	1.1	1.5	1.1
	***310.8***	1.2	0.6	0.7	0.9	1.0	0.1	0.9	1.3	1.1	1.1	1.5	0.9	0.8	0.9	1.3	1.0
*B. pertussis*	1.1	***73.9***	0.8	0.7	1.1	1.4	0.2	1.1	0.5	**3.1**	1.2	0.6	1.3	1.0	1.3	1.0	1.1
	1.0	***78.6***	0.6	0.6	0.7	1.1	0.1	0.8	0.6	2.8	1.1	0.5	1.0	0.9	1.2	0.8	1.0
*C. pneumoniae*	1.2	1.6	***95.6***	0.8	1.1	1.2	0.1	2.6	0.6	1.2	1.2	0.6	1.1	1.0	1.2	1.1	1.1
	1.2	1.8	***96.8***	0.6	1.1	1.3	0.1	2.4	0.6	1.2	1.1	0.4	1.1	1.2	1.2	1.1	1.0
*Escherichia coli*	1.2	0.4	0.5	***24.2***	0.6	1.3	0.1	0.8	0.6	1.0	1.1	1.0	1.0	0.8	1.3	0.9	1.0
	1.1	0.3	0.5	***24.1***	0.5	1.0	0.1	0.8	0.4	1.1	1.2	1.1	1.0	0.9	1.4	0.8	1.0
*H. influenzae* type a	1.6	0.3	0.6	1.0	***156.0***	1.9	0.2	0.8	1.8	1.5	1.4	1.5	1.4	1.3	1.8	1.8	1.1
	1.3	1.8	0.5	0.2	***146.3***	1.5	0.1	0.8	0.2	1.3	1.1	0.2	1.0	1.1	1.3	0.8	1.0
*H. influenzae* type b	1.1	0.9	1.2	1.6	***84.5***	***85.9***	0.1	0.8	0.3	1.1	1.3	0.8	1.0	1.0	1.1	0.6	1.1
	1.4	1.1	0.7	0.9	***98.4***	***89.1***	0.1	0.8	0.3	1.2	1.3	0.6	1.1	1.1	1.2	0.6	1.1
*Klebsiella pneumoniae*	1.2	0.3	0.3	0.5	0.4	1.5	***33.9***	1.1	0.5	1.1	1.3	0.6	1.2	0.9	1.2	0.9	1.0
	1.2	0.7	1.4	0.4	0.5	1.3	***34.3***	0.9	0.6	1.1	1.2	0.4	1.2	0.9	1.2	0.9	1.0
*L. pneumophila*	1.1	1.2	0.6	1.5	0.5	1.3	0.1	***27.9***	1.1	1.0	1.1	1.1	1.0	1.2	1.3	0.8	1.0
	0.9	0.2	0.7	0.1	0.2	0.9	0.0	***27.8***	1.5	0.9	1.1	0.1	0.8	0.7	0.7	0.3	1.0
*Moraxella catarrhalis*	1.1	1.0	0.7	0.3	0.9	1.5	0.2	0.8	***17.7***	1.3	1.2	0.2	1.0	0.8	1.2	0.5	0.9
	1.7	1.8	0.7	0.8	1.1	1.8	0.2	0.9	***18.9***	1.4	1.4	1.6	1.5	1.3	1.9	0.7	1.2
*M. pneumoniae*	1.3	1.8	0.6	0.4	1.2	1.3	0.1	0.8	0.6	***113.3***	1.3	0.3	1.2	1.1	1.4	0.9	1.1
	1.6	1.9	0.7	0.4	1.2	1.7	0.1	1.0	0.6	***111.2***	1.4	0.4	1.3	1.1	1.7	0.9	1.2
*M. tuberculosis*	1.0	0.2	1.2	0.4	1.2	1.8	0.1	1.1	1.0	1.4	***61.7***	0.3	1.4	1.2	1.5	1.3	1.1
	1.3	0.2	0.8	0.4	1.8	**2.1**	0.1	1.2	0.5	1.7	***68.2***	0.4	1.7	1.3	1.9	0.8	1.1
*Neisseria meningitidis*	0.9	1.0	0.7	1.1	0.6	1.2	0.1	0.7	0.9	1.2	1.0	***60.7***	1.1	0.7	0.8	1.2	0.8
	1.5	0.4	0.7	0.4	0.7	1.4	0.1	0.9	0.4	1.2	1.4	***73.2***	1.4	1.1	1.6	0.7	1.1
*Pseudomonas aeruginosa*	1.6	0.5	1.1	0.5	1.1	1.6	0.1	1.0	0.5	1.3	1.4	0.4	***145.1***	1.1	1.5	0.8	1.1
	1.6	0.5	0.7	0.4	1.1	1.4	0.1	0.9	0.6	1.3	1.3	0.3	***151.7***	1.2	1.4	0.8	1.2
*Staphylococcus aureus*	0.9	0.5	0.6	0.7	0.5	1.0	0.1	0.8	0.4	1.3	1.3	0.3	1.1	***106.7***	1.2	1.4	1.0
	1.1	2.7	0.5	1.0	0.6	0.8	0.1	0.9	0.3	1.6	1.1	4.0	1.0	***94.0***	1.1	1.4	1.0
*Stenotrophomonas maltophilia*	1.8	0.4	0.9	1.6	0.6	1.1	0.1	1.3	0.5	1.6	1.5	0.8	1.5	1.4	***22.5***	1.0	1.2
	0.9	0.3	0.3	0.1	0.3	1.1	0.0	0.7	1.5	0.9	1.0	0.1	0.8	0.7	***16.1***	0.3	1.0
*Streptococcus pneumoniae*	1.6	1.3	0.8	1.9	0.8	1.8	0.5	1.4	0.9	1.3	1.4	0.5	1.4	1.2	1.7	***36.6***	1.1
	1.4	2.2	0.7	1.9	0.8	1.6	0.5	1.3	0.9	1.3	1.4	0.4	1.4	1.1	1.4	***38.0***	1.1
*Streptococcus pyogenes*	1.6	0.4	1.2	0.6	1.1	0.7	0.1	1.2	0.7	1.4	1.3	0.9	1.4	1.4	1.8	1.0	***28.6***
	1.7	0.4	1.2	0.3	1.1	0.9	0.1	1.2	1.3	1.4	1.5	0.4	1.5	1.5	1.6	0.9	***35.0***
Positive cutoff	**2.3**	**8.0**	**2.0**	**2.0**	**5.0**	**2.0**	**2.0**	**3.0**	**2.0**	**3.0**	**2.0**	**5.0**	**2.2**	**2.0**	**2.0**	**2.5**	**2.0**

^*a*^ Nucleic acids from reference strains or synthetic oligonucleotides of target bacteria were tested in duplicate at 10⁵ copies per reaction using the PPID assay for specificity analysis. Results are expressed as SNRs. SNRs exceeding the cutoff values are shown in bold. Samples in which both replicates exceed the cutoff are interpreted as positive and are displayed in italics.

**TABLE 2:** Analytical specificity of the PPID assay for viral targets^*a*^

	Probe set									
Template	InfA	InfB	Rhino	RSVA	RSVB	HPIV1	HPIV2	HPIV3	HPIV4	SCV2
Influenza A virus	***583.9***	1.1	0.9	0.9	0.8	0.6	1.0	1.1	1.2	1.1
	***571.4***	0.8	0.9	0.8	0.8	0.6	1.3	0.9	0.9	1.1
Influenza B virus	1.3	***344.3***	0.9	0.8	1.1	0.4	1.1	1.1	0.6	1.5
	1.2	***352.6***	0.8	0.8	1.1	5.0	1.1	1.3	0.7	1.9
Rhinovirus	1.3	0.7	***81.4***	0.4	0.8	1.2	1.1	1.1	1.2	1.0
	1.3	1.1	***79.4***	0.4	0.9	0.2	1.3	1.2	1.2	1.0
Respiratory syncytial virus type A	1.3	0.7	0.9	***564.0***	4.0	5.8	1.3	1.4	1.5	0.9
	1.2	1.7	1.0	***545.4***	4.0	5.0	1.3	1.5	1.4	0.8
Respiratory syncytial virus type B	1.2	0.9	0.9	1.1	***824.6***	5.6	1.1	1.4	1.2	1.5
	1.4	1.0	0.9	1.8	***810.5***	5.7	1.1	1.4	1.2	1.6
Human parainfluenza virus type 1	1.2	1.2	1.1	1.6	0.8	***51.3***	1.0	1.6	1.1	1.3
	1.0	0.6	0.9	0.4	0.9	***54.6***	0.9	1.4	1.0	0.9
Human parainfluenza virus type 2	1.2	0.9	0.8	1.0	1.0	2.5	***1,018.7***	1.3	1.4	1.3
	1.3	1.9	0.8	0.4	0.8	0.5	***1,030.6***	1.2	1.2	1.0
Human parainfluenza virus type 3	1.1	0.4	1.0	1.4	0.8	1.4	1.3	***491.8***	0.7	0.9
	1.3	0.8	1.0	2.5	0.9	1.6	1.3	***477.5***	2.1	0.9
Human parainfluenza virus type 4	1.2	1.0	1.0	0.6	1.0	0.2	1.2	1.3	***1,197.3***	1.2
	1.3	0.7	1.1	0.6	0.9	0.8	1.2	1.4	***1,186.8***	1.1
SARS-CoV-2	1.2	4.4	0.9	0.4	0.9	0.2	1.0	1.2	1.2	***991.4***
	1.3	4.2	0.9	0.4	1.1	0.2	1.0	1.2	0.9	***985.8***
Positive cutoff	**10**	**15**	**3**	**5**	**2**	**10**	**2**	**2**	**12**	**5**

^*a*^ Synthetic oligonucleotides of target viruses were tested in duplicate at 10⁵ copies per reaction using the PPID assay for specificity analysis. Results are expressed as SNRs. SNRs exceeding the cutoff values are shown in bold. Samples in which both replicates exceed the cutoff are interpreted as positive and are displayed in italics.

### Assessment of analytical performance

#### Analytical specificity

To evaluate analytical specificity, total nucleic acids from reference strains and synthetic oligonucleotides for 17 bacteria and 10 viruses were tested. Additionally, to investigate cross-reactivity, nucleic acids from five strains of respiratory flora (*Haemophilus parainfluenzae* ATCC 33392, *Neisseria sicca* ATCC 9913, *Corynebacterium pseudodiphtheriticum* ATCC 10700, *Staphylococcus epidermidis* ATCC 12228, *Staphylococcus hominis* ATCC 27844) and seven non-target bacterial strains (*Salmonella typhi* ATCC 167, *Vibrio cholera* ATCC 9458, *Vibrio parahaemolyticus* ATCC 17802, *Clostridium difficile* ATCC 9689, *Clostridium perfringens* TCDC.cp3-2, *Campylobacter jejuni* TCDC.WHO C16.1, and *Bacillus cereus* TCDC.011d13—the last three strains were obtained from Taiwan Centers for Disease Control) were analyzed. All oligonucleotides and nucleic acids from a single extraction were suspended in 10 mM Tris buffer (pH 8.0) and tested at 10⁵ copies per reaction, in duplicate.

#### Analytical sensitivity

The analytical sensitivity for each target pathogen was assessed using positive controls from a single extraction, diluted in 10 mM Tris buffer (pH 8.0) to 5–10⁵ copies per reaction and tested in triplicate within a single run. The minimal detectable amount for each target was defined as the lowest amount at which all three replicates yielded positive results with SNR values exceeding the predefined target-specific cutoff.

#### Precision testing

The positive control nucleic acid from each reference strain was diluted in 10 mM Tris buffer (pH 8.0) to create moderate-to-high and low concentrations. The two concentrations were tested in duplicate on the PPID assay over 3 separate days (days 1, 2, and 3) and by different operators to evaluate its reproducibility.

### Evaluation of clinical performance

#### Interference testing

Four ETA specimens, after the pretreatment for respiratory specimens, were spiked with either 78 µM or 155 µM hemoglobin (Creative Life Sciences, Taipei, Taiwan) or with 10% or 15% (vol/vol) of a commercial nasal spray containing 0.5 mg/mL oxymetazoline hydrochloride (Siuguan, Chiayi, Taiwan). Nucleic acid was extracted and subsequently analyzed using the PPID assay.

#### Clinical performance of the PPID assay

Nucleic acids extracted from the 135 ETA specimens were tested in duplicate using the PPID assay within a single run. The data of each specimen were analyzed following the interpretation rule, and the final results were reported as either positive for target pathogens (probe set) or ND.

#### Agreement analysis between PPID assay and MC methods

Within the scope of the 12 pathogens that are the targets of the PPID assay and also typically cultured using MC methods (“common targets”), the assay results from the two methods are compared for each of all 135 specimens and categorized as follows:

1. Full agreement: PPID and MC agree on the detection or non-detection for common targets in the specimen.
2. Partial agreement (PPID > MC): PPID detects all MC-positive targets and more, with at least one detected by both methods.
3. Partial agreement (PPID ≸ MC): PPID and MC agree on at least one positive, and each method detects at least one target not detected by the other.
4. Partial agreement (MC > PPID): MC detects all PPID-positive targets and more, with at least one detected by both methods.
5. No agreement (PPID > MC): At least one target is detected by PPID, and none by MC.
6. No agreement (PPID ≸ MC): Each method detects at least one target, but none is detected by both.
7. No agreement (MC > PPID): At least one target is detected by MC, and none by PPID.

#### Singleplex quantitative PCR (qPCR) confirmation

In cases where the PPID assay detected more target pathogens or identified pathogens that were not cultured by the MC methods, the clinical specimens were further tested using singleplex qPCR for confirmation. The Materials and Methods for the singleplex qPCR analysis are provided in the Supplemental Materials and Methods Section and Table S11.

#### Clinical accuracy of the PPID assay

The clinical accuracy of the PPID assay was evaluated by using the MC methods as the comparator. The PPID and MC results were compared over the common targets. The positive percent agreement (PPA), negative percent agreement (NPA), and overall percent agreement (OPA) between the PPID and MC methods were calculated using an online statistical tool, 2 × 2 Contingency Calculator (https://tools.westgard.com/two-by-two-contingency.shtml). The results are reported as percentages with 95% confidence intervals.

### Statistics

Continuous variables are shown as means, medians, and ranges, and categorical variables are presented as frequencies and percentages. Comparative analysis was performed by χ^2^ test using GraphPad Prism v.10 (San Diego, CA, USA). A *P*-value <0.05 was considered statistically significant.

## RESULTS

### Development of the PPID assay

**Fig 1:**
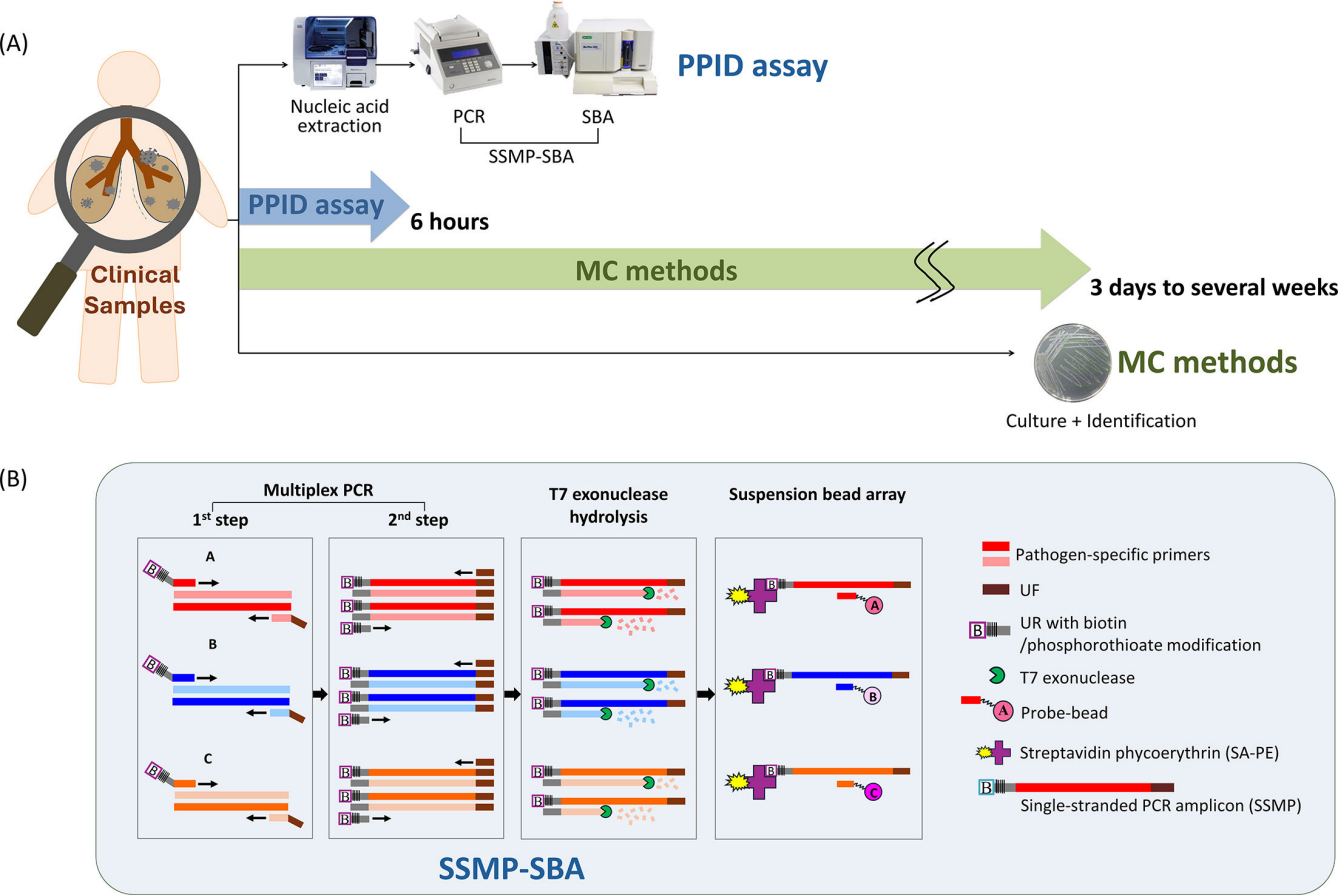
A summary of the PPID assay. (**A**) The workflow of pathogen detection in patients with pneumonia using the PPID assay and MC methods. The PPID assay detects bacterial and viral pathogens within 6 hours, whereas MC methods require several days to identify the etiology of pneumonia. For fastidious organisms, such as *M. tuberculosis*, culture-based detection may take several weeks or even exceed 1 month. (**B**) Schematic diagram of the SSMP-SBA technology. It consists of a two-step multiplex PCR amplification, generation of single-stranded PCR amplicons via T7 exonuclease hydrolysis, and subsequent detection using specific probe-coupled magnetic beads in the SBA system.

We developed the PPID assay based on the SSMP-SBA technology, enabling the simultaneous detection of 17 bacteria and 10 viruses associated with pneumonia or severe infections. Specific primers and probes were designed for detection (Table S1). The workflow of the PPID assay and the SSMP-SBA core technology are illustrated in Fig. 1.

#### Analytical specificity

The analytical specificity of the PPID assay was evaluated using 10^5^ copies per reaction of positive controls, including nucleic acids and synthetic oligonucleotides of target pathogens, tested in duplicate. Specific positive signals were observed exclusively on the corresponding probe sets (Table 1 and 2). Additionally, a slightly elevated SNR value was observed for synthetic oligonucleotides of RSV-A during testing with the RSVB probe set, suggesting minimal cross-reactivity. Five strains of respiratory flora and seven non-target bacteria were tested, and no cross-reactivity was observed (Table S3), demonstrating the high specificity of this assay.

#### Analytical sensitivity

To assess the analytical sensitivity, positive controls were tested in triplicate at concentrations ranging from 5 to 10^5^ copies per reaction. Most pathogens were reliably detected at 100 copies per reaction or below, demonstrating high sensitivity (Table 3 and 4). Although certain pathogens, including *B. pertussis*, *M. catarrhalis*, *N. meningitidis*, and *S. maltophilia*, required a minimum of 500 copies per reaction for detection. Overall, the sensitivity profile remains highly satisfactory.

**TABLE 3:** Analytical sensitivity of the PPID assay for bacterial targets^*a*^

Template	10^5^	10^4^	10^3^	500	100	50	10	5	Minimal detectable amounts
	(Copies/reaction)								
Bacteria									
*A. baumannii*	3/3	3/3	3/3	3/3	3/3	3/3	3/3	3/3	5
*B. pertussis*	3/3	3/3	3/3	3/3	0/3	0/3	0/3	0/3	500
*C. pneumoniae*	3/3	3/3	3/3	3/3	3/3	1/3	1/3	0/3	100
*E. coli*	3/3	3/3	3/3	3/3	3/3	3/3	2/3	0/3	50
*H. influenzae* type a	3/3	3/3	3/3	3/3	3/3	3/3	3/3	1/3	10
*H. influenzae* type b	3/3	3/3	3/3	3/3	3/3	3/3	3/3	3/3	5
*K. pneumoniae*	3/3	3/3	3/3	3/3	3/3	3/3	3/3	2/3	10
*L. pneumophila*	3/3	3/3	3/3	3/3	3/3	3/3	1/3	0/3	50
*M. catarrhalis*	3/3	3/3	3/3	3/3	1/3	0/3	0/3	0/3	500
*M. pneumoniae*	3/3	3/3	3/3	3/3	3/3	0/3	0/3	0/3	100
*M. tuberculosis*	3/3	3/3	3/3	3/3	3/3	2/3	0/3	0/3	100
*N. meningitidis*	3/3	3/3	3/3	3/3	1/3	0/3	0/3	0/3	500
*P. aeruginosa*	3/3	3/3	3/3	3/3	3/3	2/3	2/3	1/3	100
*S. aureus*	3/3	3/3	3/3	3/3	3/3	1/3	0/3	0/3	100
*S. maltophilia*	3/3	3/3	3/3	3/3	0/3	0/3	0/3	0/3	500
*S. pneumoniae*	3/3	3/3	3/3	3/3	3/3	0/3	0/3	0/3	100
*S. pyogenes*	3/3	3/3	3/3	3/3	3/3	1/3	0/3	0/3	100

^*a*^ Nucleic acids from reference strains or synthetic oligonucleotides of target bacteria were tested in triplicate at quantities of 5–10⁵ copies per reaction using the PPID assay for sensitivity analysis. Results are expressed as positive rates (number of positive samples per total tested samples). The minimal detectable amount for each pathogen was defined as the lowest concentration at which all replicates tested positive.

**TABLE 4:** Analytical sensitivity of the PPID assay for viral targets^*a*^

Template	10^5^	10^4^	10^3^	500	100	50	10	5	Minimal detectable amounts
	(Copies/reaction)								
Viruses									
Influenza A virus	3/3	3/3	3/3	3/3	3/3	3/3	0/3	0/3	50
Influenza B virus	3/3	3/3	3/3	3/3	3/3	3/3	0/3	0/3	50
Rhinovirus	3/3	3/3	3/3	3/3	3/3	1/3	0/3	0/3	100
Respiratory syncytial virus type A	3/3	3/3	3/3	3/3	3/3	3/3	0/3	0/3	50
Respiratory syncytial virus type B	3/3	3/3	3/3	3/3	3/3	0/3	0/3	0/3	100
Human parainfluenza virus type 1	3/3	3/3	3/3	3/3	3/3	3/3	0/3	0/3	50
Human parainfluenza virus type 2	3/3	3/3	3/3	3/3	3/3	3/3	0/3	0/3	50
Human parainfluenza virus type 3	3/3	3/3	3/3	3/3	3/3	3/3	3/3	0/3	10
Human parainfluenza virus type 4	3/3	3/3	3/3	3/3	3/3	3/3	0/3	0/3	50
SARS-CoV-2	3/3	3/3	3/3	3/3	3/3	3/3	0/3	0/3	50

^*a*^ Synthetic oligonucleotides of target viruses were tested in triplicate at quantities of 5–10⁵ copies per reaction using the PPID assay for sensitivity analysis. Results are expressed as positive rates (number of positive samples per total tested samples). The minimal detectable amount for each pathogen was defined as the lowest concentration at which all replicates tested positive.

#### Precision testing

The precision of the PPID assay was evaluated using eight bacterial targets, as shown in Table S4. Across all eight bacterial targets tested, all replicates yielded consistent positive results, regardless of different operators and test days. This indicates there are no significant differences in the interpretation of the results among different users and across various time points. The PPID assay achieved 100% detection rates at both moderate-to-high and low concentrations of the target bacteria. For most nucleic acid samples, the PPID assay yielded specific positive signals only with their corresponding probe sets, demonstrating good specificity. However, a few exceptions were observed: for *B. pertussis* at 10^5^ copies per reaction, cross-reactivity was seen with the Mpn probe set. Occasional single-replicate signals slightly above the cutoff were observed for *B. pertussis* against the Eco probe set, and for *M. tuberculosis* against the Mca or Sau probe sets, both at 10^5^ copies per reaction. Despite these rare elevated signals, they were interpreted as negative according to the predefined rule requiring both replicates to exceed the cutoff. In summary, the precision testing verified the overall specificity of the PPID assay, with a few minor exceptions of cross-reactivity and occasional single-replicate elevations that did not impact the final interpretation of the results.

### Demographics and clinical characteristics of the enrolled patients

To investigate the clinical applicability of the PPID assay, we assessed its performance in pathogen detection and identification among patients with pneumonia admitted to the ED of TSGH, Taiwan, from September 2018 to September 2023. A total of 135 patients were enrolled, whose demographic and clinical statistics are outlined in Table 5. Among them, 61.5% were male (83/135), with an average age of 71.9 years (range: 24–97). CAP accounted for 74.1% (100/135) of cases, HAP for 12.6% (17/135), and HCAP for 13.3% (18/135). A majority of patients (76.3%, 103/135) had at least one underlying comorbidity. The most common comorbidities were cardiovascular disease (60%, 81/135), endocrine disorders (28.1%, 38/135; primarily diabetes), cancer (20%, 27/135), and respiratory disease (14.8%, 20/135; mainly chronic obstructive pulmonary disease).

**TABLE 5:** Demographics and baseline clinical characteristics of patients with pneumonia enrolled in this study (*N* = 135)

Characteristics	Average (median, range)	*N* (%)
Age (years)	71.9 (73, 24–97)	
Gender		
Male	NA^*f*^	83 (61.5%)
Female	NA	52 (38.5%)
Blood laboratory examination		
Procalcitonin (ng/mL)^*b*^	7.2 (1.0, 0.03–>200)	114
C reactive protein (mg/L)^*c*^	12.3 (10.4, 0.1–48.0)	86
White blood cell count (×10^9^/L)^*d*^	13.1 (10.9, 2.0–43.3)	126
Comorbidity		103 (76.3)
Respiratory disease (COPD)^*e*^	NA	20 (14.8)
Cardiovascular disease	NA	81 (60.0)
Endocrine disorders (diabetes)	NA	38 (28.1)
Cancer	NA	27 (20.0)
Renal disease	NA	1 (0.7)
Hepatic disease (liver cirrhosis)	NA	3 (2.2)
Types of pneumonia		
CAP	NA	100 (74.1%)
HAP	NA	17 (12.6%)
HCAP	NA	18 (13.3%)
Outcome		
Discharge	NA	100 (74.1%)
Expired	NA	31 (23.0%)
Critical AAD^*a*^	NA	4 (3.0%)

^*a*^ Critical AAD, against advice discharge under critical condition.

^*b*^ Information was obtained from 114 patients.

^*c*^ Information was obtained from 86 patients.

^*d*^ Information was obtained from 126 patients.

^*e*^ COPD, chronic obstructive pulmonary disease.

^*f*^ NA, not applicable.

### Evaluation of clinical performance

#### Interference testing

To evaluate the potential influence of interfering substances on PPID performance, hemoglobin and nasal spray were chosen as representative endogenous and exogenous substances, respectively. Under the condition of 155 µM hemoglobin, all four spiked ETA specimens yielded “Not Detected” results in the PPID assay, indicating complete inhibition of pathogen detection. At a concentration of 78 µM hemoglobin, inhibition was observed in only one specimen (#025). In contrast, spiking with the nasal spray at 10% or 15% (vol/vol) exhibited no inhibitory effect on the assay results (Table S5).

#### Pathogen detection using the PPID assay

**Fig 2:**
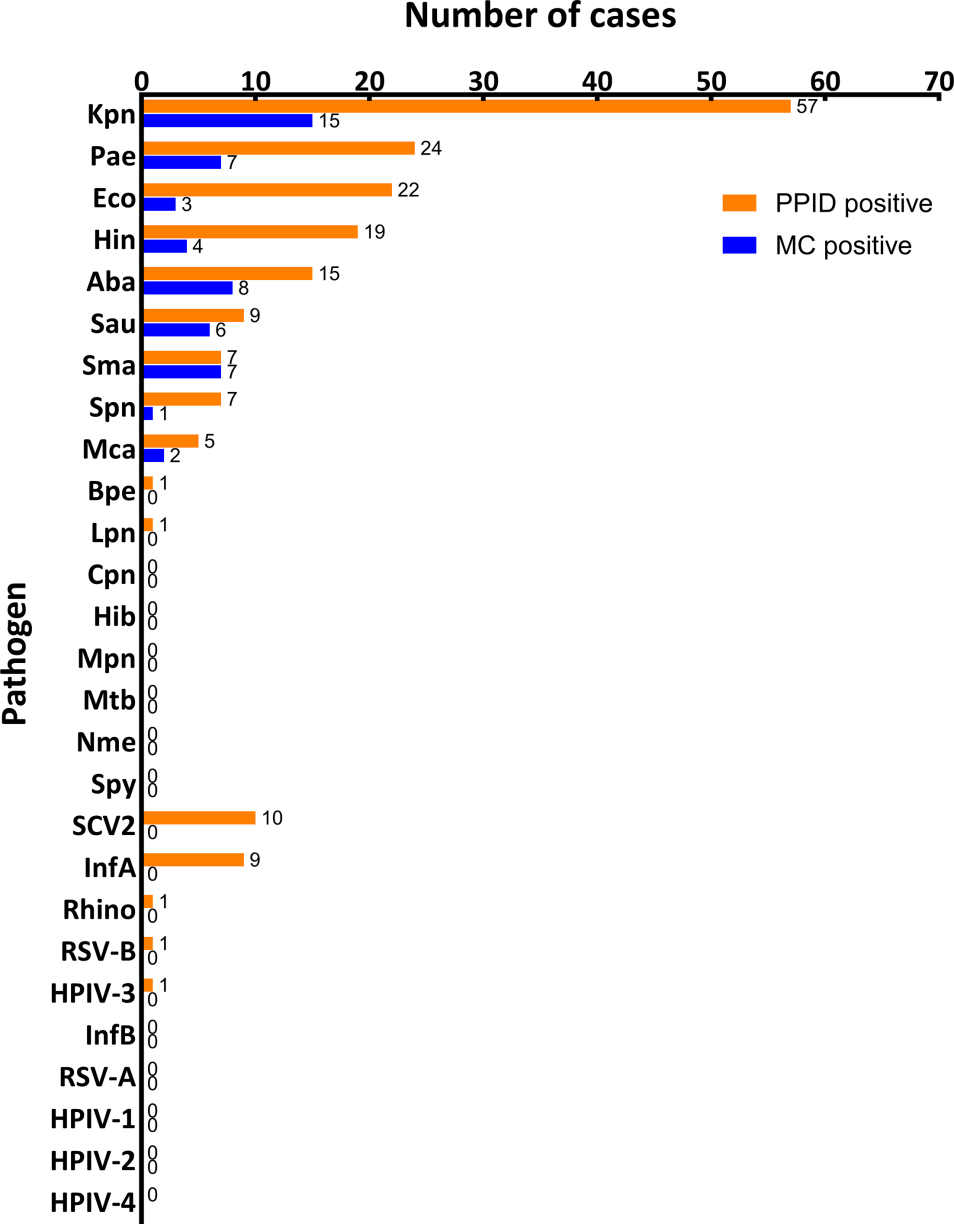
Distribution of pathogens identified in patients with pneumonia (*N* = 135) using the PPID assay (orange bars) and MC methods (blue bars). The number of positive samples for each pathogen is indicated. Kpn, *Klebsiella pneumoniae*; Pae, *Pseudomonas aeruginosa*; Eco, *Escherichia coli*; Hin, *Haemophilus influenzae*; Aba, *Acinetobacter baumannii*; Sau, *Staphylococcus aureus*; Sma, *Stenotrophomonas maltophilia*; Spn, *Streptococcus pneumoniae*; Mca, *Moraxella catarrhalis*; Bpe, *Bordetella pertussis*; Lpn, *Legionella pneumophila*; Cpn, *Chlamydia pneumoniae*; Hib, *Haemophilus influenzae* type B; Mpn, *Mycoplasma pneumoniae*; Mtb, *mycobacterium tuberculosis*; Nme, *Neisseria meningitidis*; Spy, *Streptococcus pyogenes*; SCV2, SARS-CoV-2; InfA, influenza A virus; Rhino, rhinovirus; RSV-B, respiratory syncytial virus type B; HPIV-3, human parainfluenza virus type 3; InfB, influenza B virus; RSV-A, respiratory syncytial virus type A; HPIV-1, human parainfluenza virus type 1; HPIV-2, human parainfluenza virus type 2; HPIV-4, human parainfluenza virus type 4.

In the ETA specimens from the 135 cases, the PPID assay identified pathogens in 77.8% (105/135) of them. Among which, bacteria accounted for 79.0% (83/105), viruses for 15.2% (16/105), and mixed bacterial and viral infections for 5.7% (6/105) (Table 6 and Table S6). The five leading pathogens identified by the PPID assay were *K. pneumoniae* (42.2%, 57/135), *P. aeruginosa* (17.8%, 24/135), *E. coli* (16.3%, 22/135), *H. influenzae* (14.1%, 19/135), and *A. baumannii* (11.1%, 15/135) (Fig. 2).

**TABLE 6:** Pathogen detection in patients with pneumonia using the PPID assay, MC methods, and a combination of both methods^*a*^

Pathogen detection, *N* (%)		PPID	MC	PPID + MC
Positive‍‍^*b*^		105 (77.8)	61 (45.2)	116 (85.9)
	Bacteria	83	61	NA
	Viruses	16	NA	NA
	Bacteria and viruses	6	NA	NA
ND‍‍^*c*^		30 (22.2)	74 (54.8)	19 (14.1)

^*a*^ ND, not detected; NA, not applicable.

^*b*^ Significant differences were observed in the pathogen-positive rates between the PPID assay and MC methods (*P* < 0.0001), and between MC methods and combined detection (*P* < 0.0001). However, no significant difference was found between the PPID assay and combined detection (*P* = 0.0824).

^*c*^ In the cases of the MC methods, ND refers to cultures showing no growth, normal pharyngeal flora, or mixed flora.

Notably, multiple pathogens were detected in 48.6% (51/105) of positive cases (Table 7), with *K. pneumoniae*, *P. aeruginosa*, and *E. coli* being the most common pathogens among multiple detections (Table S7). Co-detections of bacterial and viral pathogens were observed in six specimens (specimen no. 049-051, 133-135 in Table S6).

**TABLE 7:** Multiple pathogen detection in patients with pneumonia using the PPID assay and MC methods^*a*^

Pathogen detection	*N* (%)			
	PPID		MC	
ND	30		74	
Pathogen-positive	105		61	
Single pathogen	54 (51.4)^*b*^		40 (65.6)^*c*^	
1 Bacteria		38 (36.2)^*b*^		40 (65.6)^*c*^
1 Virus		16 (15.2)^*b*^		NA
Multiple pathogens	51 (48.6)^*b*^		21 (34.4)^*c*^	
2 Bacteria		26 (24.8)^*b*^		11 (18.0)^*c*^
3 Bacteria		12 (11.4)^*b*^		9 (14.8)^*c*^
4 Bacteria		5 (4.8)^*b*^		1 (1.6)^*c*^
5 Bacteria		2 (1.9)^*b*^		0
1 Virus + 1 bacteria		2 (1.9)^*b*^		NA
1 Virus + 2 bacteria		3 (2.9)^*b*^		NA
1 Virus + 3 bacteria		1 (1.0)^*b*^		NA

^*a*^ ND, not detected. NA, not applicable.

^*b*^ The percentages are calculated using PPID assay-positive cases (*N* = 105) as the denominator.

^*c*^ The percentages are calculated using MC method-positive cases (*N* = 61) as the denominator.

#### Comparison of detection rates and pathogen profiles between the PPID assay and MC methods

The PPID assay detected pathogens in 77.8% of specimens, compared to 45.2% (61/135) detected by the MC methods. Combining the results from both methods increased the overall detection rate to 85.9% (Table 6). Furthermore, the PPID assay identified multiple pathogens in 48.6% (51/105) of pathogen-positive cases, whereas the MC methods detected co-infections in only 34.4% (21/61) of positive specimens (Table 7).

#### Agreement analysis between PPID assay and MC methods

The clinical performance of the PPID assay is evaluated by comparing those of MC methods using a total of 135 respiratory specimens as shown in Table S6. The agreement between the two methods is classified into seven categories, listed in Table 8 and Tables S6 and S8. Please refer to the subsection “Agreement analysis between PPID assay and MC methods” in Materials and Methods for detailed classification criteria. Full agreement is observed for 56 specimens. Partial agreement includes PPID > MC for 16 specimens, PPID ≸ MC for 2 specimens, and MC > PPID for 1 specimen. The cases of disagreement, in which no pathogen is detected by both methods in each specimen, include PPID > MC for 47 specimens, PPID ≸ MC for 6 specimens, and MC > PPID for 7 specimens (Table 8).

**TABLE 8:** Agreement analysis between PPID assay and MC methods

Agreement category	Number, *N*	Percentage, %^*a*^
Full agreement	56	41.5
Partial agreement (PPID > MC)	16	11.9
Partial agreement (PPID ≸ MC)	2	1.5
Partial agreement (MC > PPID)	1	0.7
No agreement (PPID > MC)	47	34.8
No agreement (PPID ≸ MC)	6	4.4
No agreement (MC > PPID)	7	5.2
Total	135	100.0

^*a*^ The percentages are calculated using the total of 135 ETA specimens as the denominator.

#### Clinical accuracy of the PPID assay

The clinical accuracy of the PPID assay was evaluated by using the MC methods as the comparator. The results are summarized in Table S12. For most respiratory bacteria targets, the PPID assay demonstrates high PPA, NPA, and OPA compared to the MC methods. Specifically, the PPID assay shows 100% PPA, 85.6% NPA, and 85.9% OPA for *E. coli*. Similarly, for *S. pneumoniae*, the PPID assay has 100% PPA, 95.5% NPA, and 95.6% OPA. However, there are a few exceptions. For *H. influenzae*, *S. aureus*, *M. catarrhalis,* and *A. baumannii*, the PPA is 50.0%. Additionally, for *S. maltophilia*, the PPA is 42.9%, suggesting a substantial number of false-negative results with the PPID assay.

These findings suggest that the PPID assay generally performs well in detecting the majority of the respiratory bacteria targets, but there are a small number of specimens for which the assay exhibited false negatives, assuming the MC method as the comparator.

## DISCUSSION

We developed a fast and sensitive pneumonia pathogen detection assay, namely the PPID assay. This readily expandable 27-plex assay can simultaneously identify 17 bacteria and 10 viruses in 96 samples within 6 hours. Demonstrating exceptional analytical performance, the assay reliably detects 5–100 copies per reaction for most pathogens. Clinical data from patients with pneumonia or severe respiratory infections in EDs show that the PPID assay achieves a pathogen detection rate of 77.8%. Combining the PPID assay with MC methods raises detection rates to 85.9%, demonstrating its potential to complement and enhance conventional diagnostics. The PPID assay is based on the SSMP-SBA platform, which combines PCR amplification with SBA technology, a magnetic bead-based method for multiplex detection. Compared to qPCR, which typically detects four to six targets per reaction due to fluorescence channel limitations, SBA enables simultaneous detection of up to 100–500 targets (22). Unlike microarrays that rely on solid-phase hybridization and require 8–24 hours (23), SBA uses liquid-phase hybridization for faster kinetics, lower background, and shorter turnaround times (4–6 hours) (24). Compared to other current multiplex panels, this integrated platform allows the PPID assay to provide rapid, scalable, and flexible detection of pneumonia pathogens, with potential for future customized expansion.

The PPID assay offers notable advantages over other nucleic acid amplification test-based multiplex diagnostics. Unlike most commercial assays, which primarily target respiratory viruses and atypical bacteria, the PPID assay simultaneously detects key respiratory pathogens including *H. influenzae* type b, *B. pertussis*, *N. meningitidis*, and *S. maltophilia*. This broader coverage aligns more effectively with the clinical needs for rapid differential diagnosis and treatment guidance, particularly in emergency pneumonia cases. In terms of throughput, the PPID assay can process up to 96 samples within 6 hours, whereas the FilmArray Respiratory/Pneumonia Panel (bioMérieux, France) is limited to one sample in 1–1.5 hours per instrument, and the NxTAG Respiratory Pathogen Panel (NxTAG RPP) (Diasorin, Antony, France) processes 48 samples in 4 hours (25–27). This high-throughput capacity makes the PPID assay ideal for large-scale screening during outbreaks and crucial for respiratory infectious disease surveillance. Both the NxTAG RPP and the PPID assay utilize the Luminex xMAP bead-based multiplex technology for pathogen detection (22, 28). However, the NxTAG RPP employs anti-TAG magnetic beads to detect TAG-labeled PCR products using 24-base DNA sequences unrelated to target pathogens. In contrast, the PPID assay uses pathogen-specific probes on magnetic beads to detect single-stranded pathogen-specific PCR products, achieving superior precision through targeted PCR amplification and probe detection.

In the precision testing (Table S4), the observed cross-reactivity of *B. pertussis* at high concentrations (10^5^ copies per reaction) with the Mpn probe set suggests that the assay may have some limitations in differentiating between these two bacterial species when present at high levels. This cross-reactivity could lead clinicians to mistakenly believe the patient is co-infected with *B. pertussis* and *M. pneumoniae*, resulting in unnecessary treatment measures and potentially triggering unwarranted preventive actions, such as isolation and prophylactic antibiotic therapy for close contacts. To address this issue, we reiterate the importance of carefully interpreting PPID results following the stringent rules, especially when dealing with samples containing high pathogen loads, and also recommend that, when *B. pertussis* and *M. pneumoniae* are both detected by the PPID, the patient’s clinical presentation should be carefully evaluated: if it is not consistent with *M. pneumoniae* infection, false-positive results due to cross-reactivity should be suspected. Consider using alternative diagnostic methods, such as PCR, to directly detect the presence of *M. pneumoniae* and verify the PPID results. Similarly, the observed occasional single-replicate signals slightly above the cutoff for *B. pertussis* against the Eco probe set, and for *M. tuberculosis* against the Mca or Sau probe sets, also show the interpretation rules as effective in avoiding false-positive reporting, demonstrating the assay’s ability to maintain specificity even in the face of these rare elevated signals.

The precision testing for the specificity of the PPID assay verified its overall performance in accurately detecting the targeted organisms reliably. This is a critical performance characteristic, as cross-reactivity results could lead to false-positive results, which in turn could result in misdiagnosis and inappropriate treatment decisions. Specifically, cross-reactivity occurs when the PPID assay reacts with non-target organisms, causing the test to incorrectly identify the presence of a pathogen that is not actually there. This false-positive result may prompt the clinician to prescribe antimicrobial therapy that is not appropriate for the patient’s actual condition. The use of inappropriate antimicrobial therapy driven by cross-reactivity could contribute to the development and spread of antibiotic-resistant pathogens. Therefore, confirming the PPID assay results in conjunction with clinical symptoms and other diagnostic methods is necessary.

Interference from complex clinical matrices, such as ETA specimens, is a critical consideration for molecular diagnostic assays, including the PPID. We assessed the effects of hemoglobin and nasal spray on the clinical performance of the PPID assay. As shown in Table S5, high concentrations of hemoglobin (155 µM) completely inhibited pathogen detection in all tested ETA samples, while moderate levels (78 µM) caused partial inhibition in one *H*. *influenzae*-positive specimen. These results align with previous studies that recognize hemoglobin as a significant PCR inhibitor, particularly when present in high concentrations or inadequately removed during extraction (29, 30). Therefore, the blood-contaminated samples should be interpreted with caution or reprocessed, especially at low pathogen loads.

The agreement analysis demonstrated comparative performance of the PPID assay and the MC methods in detecting the 12 common targets as shown in Table 8; Tables S6 and S8. Out of the 135 respiratory specimens tested, the PPID assay exhibited full agreement with the MC method for 56 samples, indicating the PPID assay’s ability to accurately detect respiratory pathogens with results matching those of the traditional approach.

Even in situations where the two methods do not fully agree with each other, the PPID assay still demonstrated good performance. In 63 samples—including 16 cases of partial agreement (PPID > MC) and 47 cases of no agreement (PPID >MC)—the PPID assay not only confirmed all the MC-positive results but also detected additional pathogens typically covered by MC. The difference could be attributed to prior antibiotic treatment in the study subjects, leading to negative bacterial cultures; or the MC results may have been interpreted as mixed flora or normal pharyngeal flora and did not reveal the specific pathogens; or to the potentially higher sensitivity of the PPID over the MC methods for certain pathogens.

On the other hand, in 16 samples—including seven cases of no agreement (MC > PPID) and six cases of no agreement (PPID ≸ MC), two cases of partial agreement (PPID ≸ MC), and one case of partial agreement (MC > PPID)—the MC methods detected pathogens that were not identified by the PPID assay. This suggests that in some cases, the MC methods may be more sensitive to certain pathogens.

Facing the lack of culture for atypical, fastidious bacteria and viruses in the MC methods, the PPID assay was able to identify additional targets, such as SARS-CoV-2, influenza A, human parainfluenza virus type 3 (HPIV-3), rhinovirus, RSV-B, *B. pertussis*, and *L. pneumophila* (as shown in Table S9). The analysis suggests that using the PPID and MC methods in combination can provide a more comprehensive assessment of respiratory infections.

Compared to previous studies on pathogen detection in severe pneumonia (including CAP, HAP, and HCAP), which typically focused on specific pathogens (31–33), the PPID assay allows for the simultaneous detection of multiple pathogens, including bacteria and viruses, as shown in Table S7, thus providing more comprehensive microbiological information. Additionally, the high sensitivity of the PPID assay enables the detection of a broader range of microorganisms, including less common pathogens such as *L. pneumophila* (34–37), which is a critical cause of CAP and HAP and has been observed to surge in cases, particularly post-COVID-19 (38–40). In Taiwan, a total of 1,124 confirmed cases were reported in 2024, compared to 413 cases in 2023, showing a 2.7-fold increase (41, 42). The challenge in accurately diagnosing *L. pneumophila* infections lies in the fact that this pathogen is an atypical bacterium, often overlooked by routine culture methods used in clinical laboratories. Instead, the detection typically relies on *L. pneumophila* antigen testing in urine specimens, which can delay the diagnosis and treatment. In this study, the PPID assay was able to detect *L. pneumophila* in one case (specimen no. 099 in Table S6), which was subsequently confirmed by singleplex qPCR. This finding emphasizes the potential of the PPID assay as a rapid pathogen screening tool for critically ill emergency patients, providing a valuable reference for diagnosis and guiding subsequent confirmatory testing, such as cultures or other methods.

Multiple infections are common in infectious diseases, particularly respiratory infections, and are associated with adverse patient outcomes, including prolonged intensive care unit stays and increased mortality (43–47). Bacterial-viral co-infections are of particular concern, with previous studies focusing primarily on bacterial co-infections with influenza viruses or RSVs (48–51). The PPID assay, being a multiplex detection platform capable of simultaneously identifying bacterial and viral pathogens, offers a significant advantage in rapidly identifying co-infection profiles at the early stages of hospitalization. In this study, the PPID assay identified more polymicrobial infections and detected six bacterial-viral co-infections involving influenza A virus, HPIV-3, and SARS-CoV-2. These findings highlight its potential to facilitate prompt and precise treatment and infection control strategies.

The emergence of next-generation sequencing and culture-independent identification techniques has demonstrated that a significant number of infections, if not the majority, are either polymicrobial in origin or presentation. Such polymicrobial infections frequently correlate with heightened severity of infection and worse outcomes for patients. The higher polymicrobial detection rate observed may reflect both the increased sensitivity of molecular methods and the inherent limitation of multiplex pneumonia panels in distinguishing true infection from colonization. While the high sensitivity of the PPID assay allows for the detection of a wide array of microorganisms, including pathogens and commensals, accurately interpreting the clinical significance of these findings poses a significant challenge. Misidentifying commensals as true pathogens may lead to unnecessary or inappropriate antibiotic treatments, potentially exacerbating the development of resistance and resulting in adverse outcomes for patients. Conversely, failing to recognize the potential clinical relevance of certain polymicrobial infections may result in suboptimal treatment and poorer patient prognosis (52, 53). This challenge is particularly pertinent to molecular testing methods that detect commensals (54, 55).

To address this issue, further efforts are needed to establish the correlation between PPID assay results and clinical manifestations, as well as underlying diseases, in order to differentiate between clinically significant pathogens and commensals (56, 57). Additionally, complementing the PPID assay with other diagnostic methods, such as quantitative PCR or targeted cultures, may enhance our understanding of the detected microorganisms and their importance in the clinical context (58, 59).

In the accuracy study, as shown in Table S12, the overall results demonstrate that the PPID assay has good clinical accuracy compared to the MC methods for the majority of the respiratory bacteria targets. The high PPA, NPA, and OPA values indicate that the PPID assay can reliably detect and differentiate the targeted pathogens in clinical samples. The exceptions observed for *H. influenzae*, *A. baumannii*, and *S. maltophilia* suggest that there may be room for the PPID assay to improve in accurately identifying these specific bacteria. Overall, the results provide valuable insights into the clinical performance of the PPID assay and highlight the importance of continuous evaluation and optimization to ensure accurate and reliable pathogen detection, especially for the challenging bacterial targets.

*K. pneumoniae* is a major causative pathogen of pneumonia worldwide, including in Taiwan (60–62). In this study, *K. pneumoniae* was the most frequently detected pathogen in the PPID assay. Moreover, *K. pneumoniae* was the predominant pathogen in cases of multiple infections, possibly due to its propensity to infect immunocompromised individuals or those with underlying health conditions. Besides pneumonia, *K. pneumoniae* is involved in various infections, including urinary tract infections, liver abscesses, meningitis, and bacteremia, contributing to increased morbidity and mortality (63–65). While this study is focused on respiratory specimens, the flexible design of the PPID assay supports future development of syndromic testing for infections at other anatomical sites. Such applications, however, will require separate validation to ensure diagnostic performance for additional specimen types and pathogens.

*S. maltophilia*, an opportunistic pathogen, is often associated with nosocomial infections and is notorious for its intrinsic multidrug resistance and elevated mortality rates (66, 67). Approximately 55% of *S. maltophilia* infections involve the respiratory tract, posing a significant threat, especially to immunocompromised patients (66). We incorporated *S. maltophilia* as a target pathogen in the PPID assay and detected seven cases of *S. maltophilia* infection in this study. Our findings also highlight the frequent co-infections involving *S. maltophilia*, particularly with *P. aeruginosa* and *K. pneumoniae*, consistent with previous reports indicating that such co-infections significantly increase mortality risks (68). The PPID assay reliably identifies *S. maltophilia* and its co-infections, addressing limitations in current bacterial identification systems, which often struggle to accurately differentiate *S. maltophilia*, increasing the risk of misdiagnoses (66).

In this study, non-PPID target pathogens were identified in 24.4% of the specimens (33/135) using the MC methods as shown in Table S10. Among these, *Candida albicans* was the most frequently detected, with a positivity rate of 10.4% (14/135), followed by other non-*C*. *albicans* yeasts (3.7%, 5/135). To broaden the clinical scope of the PPID assay, we are actively expanding it to include key respiratory-associated fungi, such as *C. albicans*, as detection targets, leveraging its high sensitivity and specificity for rapid and accurate identification. This aims to improve the timely diagnosis and treatment of fungal respiratory infections, especially in high-risk patient populations.

This study has some limitations. First, it was conducted at a single center with a limited sample size, which may not fully represent the epidemiology and distribution of respiratory pathogens in EDs across Taiwan. Larger, multi-center studies are necessary to provide a more comprehensive understanding of respiratory pathogen prevalence in this region.

Second, as a targeted detection method, the PPID assay identifies up to 27 common and clinically significant pathogens. However, its limited target coverage prevents comprehensive detection of all potential pathogens. Combining the PPID assay with traditional culture methods for pneumonia diagnosis in ED patients ensures rapid and sensitive pathogen detection, supports timely treatment decisions, and reduces the risk of missing less common pathogens.

Third, as a nucleic acid amplification test, the PPID assay is a powerful diagnostic tool for pathogen detection, offering distinct advantages over conventional MC methods, such as short turnaround time and high sensitivity. However, the assay cannot differentiate between commensals or true pathogens, viable and non-viable pathogens in a sample, nor can it distinguish active infections from residual, non-viable pathogen DNA or RNA from previously resolved infections. This limitation may result in potential misinterpretation of the infection status. Additionally, special care must be taken to minimize contamination risks from the environment or trace contaminants within samples, as these could lead to false-positive results.

Fourth, the MC methods were performed by a clinical laboratory according to the clinical demands and as necessary, and their reports were subsequently made available to us. The PPID assay was conducted on frozen specimens in batches as part of this study later. As a result, we did not have the opportunity to check the PPID assay against the cultures for commensal organisms versus true pathogens. This represents a limitation of the current study design. To fully leverage the clinical value of the PPID assay in the future, it is recommended that the interpretation of PPID results be made in conjunction with clinical symptoms, microbiological culture results, and other diagnostic methods to make informed decisions regarding patient management.

## CONCLUSION

In conclusion, the PPID assay offers a rapid, flexible, and sensitive approach to respiratory pathogen detection, effectively addressing critical clinical and epidemiological needs. Integrating the PPID assay into routine clinical practice could significantly enhance the identification of pneumonia pathogens, facilitate the early initiation of appropriate antimicrobial therapies, and potentially improve patient outcomes. Future advancements will expand the scope of this assay to include fungal pathogen detection and antimicrobial resistance markers, further strengthening its clinical utility.

## SUPPLEMENTAL MATERIAL

Supplemental material spectrum.00811-25-s0001.docx Supplemental materials and methods, and Tables S1 to S12.

ASM does not own the copyrights to Supplemental Material that may be linked to, or accessed through, an article. The authors have granted ASM a non-exclusive, world-wide license to publish the Supplemental Material files. Please contact the corresponding author directly for reuse.
