# Supplementary material for: Development and diagnostic performance of a sensitive multiplex pneumonia pathogen identification assay for emergency department patients with pneumonia
Source: Microbiol Spectr. 2025 Sep 3;13(10):e00811-25. doi: 10.1128/spectrum.00811-25 (PMC12502684; doi:10.1128/spectrum.00811-25)
Supplement: Supplemental material — s and methods, and Tables S1 to S12. [file spectrum.00811-25-s0001.docx]

**Development and diagnostic performance of a sensitive multiplex pneumonia pathogen identification assay for emergency department patients with pneumonia**

Pei‑Yi Tsui‍,‍^a‍,b^ Chia‑Wei Hong‍,‍^c^ Yi‑Da Tsai‍,‍^c^ Chu‑Yang Chien‍,^b‍,d^ Yi‑Ling Chen‍,^b‍,d^ Hsin‑Hsien Huang‍,^b^ Chin‑Mao Hung‍,^a‍,b^ Feng‑Ping Lin‍,^b^ Chung‑Chih Liang‍,^b^ Chih‑Yuan Lin‍,^e‍,f^ Shih‑Hung Tsai‍,^c‍,g‍,h^ Hui‑Ling Hsu‍^a‍,b‍,i^

^a‍‍^Graduate Institute of Medical Sciences, National Defense Medical University, Taipei, Taiwan, Republic of China

^b‍‍^Institute of Preventive Medicine, National Defense Medical University, New Taipei City, Taiwan, Republic of China

^c‍‍^Department of Emergency Medicine, Tri-Service General Hospital, National Defense Medical University, Taipei, Taiwan, Republic of China

^d‍‍^Department of Materials Science and Engineering, National Taiwan University of Science and Technology, Taipe, Taiwan, Republic of China

^e‍‍^Division of Cardiovascular Surgery, Department of Surgery, Tri-Service General Hospital, National Defense Medical University, Taipei, Taiwan, Republic of China

^f‍‍^National Defense Medical University, Taipei, Taiwan, Republic of China

^g‍‍^Taichung Armed Forces General Hospital, Taichung, Taiwan, Republic of China

^h‍‍^Department of Physiology and Biophysics, Graduate Institute of Physiology, National Defense Medical University, Taipei, Taiwan, Republic of China

^i‍‍^Department of Microbiology and Immunology, National Defense Medical University, Taipei, Taiwan, Republic of China

Address correspondence to Hui‑Ling Hsu, [hlhsu@mail.ndmctsgh.edu.tw](mailto:hlhsu@mail.ndmctsgh.edu.tw) or Shih‑Hung Tsai, [tsaishihung@yahoo.com.tw](mailto:tsaishihung@mail.ndmctsgh.edu.tw).

Hui‑Ling Hsu and Shih‑Hung Tsai contributed equally to this article. The author order was determined by consensus.

**Supplementary Material**

**Materials and Methods**

**Singleplex qPCR** (For confirmation of pathogen detection results by the PPID assay)

To confirm bacterial pathogen detection, singleplex qPCR assays were performed using either TaqMan or SYBR Green chemistry. Each 20 μl TaqMan reaction contained 4 μl of template DNA, 200 nM of pathogen-specific primers and probes, and 1× TaqMan Fast Advanced Master Mix (Applied Biosystems, Foster City, CA, USA). For *L. pneumophila*, SYBR Green–based qPCR was used, with each 20 μl reaction containing 4 μl of template DNA, 200 nM of primers, and 1× QuantiTect SYBR Green PCR Master Mix (QIAGEN, Hilden, Germany). Thermal cycling was performed on a QuantStudio 5 Real-Time PCR System (Applied Biosystems) with the following conditions: initial enzyme activation at 95°C for 2 min, flowed by 45 cycles of 95°C for 5 s and 60°C for 30 s. Melt curve analysis was performed for SYBR Green reactions.

To confirm viral pathogen detection, each 20 μL RT-qPCR reaction included 5 μl of nucleic acid template, 300 nM of primers, 200 nM of probes, and 1× TaqMan Fast Virus 1-Step Master Mix (Applied Biosystems). Cycling conditions were: reverse transcription at 50°C for 5 min, enzyme activation at 95°C for 30 s, followed by 45 cycles of 95°C for 5 s and 58°C for 30 s. Reactions were conducted on the same instrument.

**Tables**

**Table S1.** Primers and probes used in the PPID assay

| **Target** | **Probe set** | **Target gene** | **Primers & Probes‍‍*^a^*** | **Sequence‍‍*^b^* (5ʹ to 3ʹ)** |
| --- | --- | --- | --- | --- |
| *Acinetobacter baumannii* | Aba | *ompA* | UR-Aba-F | CACCTCGACCCACTCATCTTGGTGGTCACTTGAAGC |
|  |  |  | UF-Aba-R | **CCATCCAGATTCACTCC**ACTCTTGTGGTTGTGGAGCA |
|  |  |  | AM12C-Aba-P | AAGTTGCTCCAGTTGAACCAACTCCA |
| *Bordetella pertussis* | Bpe | *transposase* | UF-Bpe-F | **CCATCCAGATTCACTCC**CGCTTGCTCACCGACAAT |
|  |  |  | UR-Bpe-R | CACCTCGACCCACTCAGCCTTGCCATTGGTCTGTG |
|  |  |  | AM12C-Bpe-P | TTTCGCAGCCGCGCCTTCGC |
| *Chlamydia pneumoniae* | Cpn | *rpoB* | UF-Cpn-F | **CCATCCAGATTCACTCC**CAGCCATTATTCACCGTCGTA |
|  |  |  | UR-Cpn-R | CACCTCGACCCACTCATGCCGTTTCGTAATCTGATAGAA |
|  |  |  | AM12C-Cpn-P | GTGGATCTTTTAATGCCTAACTGTG |
| *E. coli* | Eco | *uidA* | UR-Eco-F | CACCTCGACCCACTCACGGAAGCAACGCGTAAACTC |
|  |  |  | UF-Eco-R | **CCATCCAGATTCACTCC**TGAGCGTCGCAGAACATTACA |
|  |  |  | AM12C-Eco-P | ATTGACGCAGGTGATCGGA |
| *Haemophilus influenzae* | Hin | *hpd* | UF-Hinf-F | **CCATCCAGATTCACTCC**GGTTAAATATGCCGATGGTGTTG |
|  |  |  | UR-Hinf-R | CACCTCGACCCACTCATGCATCTTTACGCACGGTGTA |
|  |  |  | AM12C-Hinf-P | TCCGTTGGTAAAAGAACTTGCAC |
| *Haemophilus influenzae* type b | Hib | *cap* | UF-Hib-F | **CCATCCAGATTCACTCC**CAAGATACCTTTGGTCGTCTGCTA |
|  |  |  | UR-Hi-R | CACCTCGACCCACTCATAGGCTCGAAGAATGAGAAGTTTTG |
|  |  |  | AM12C-Hib-P | ATGATATGGGTACATCTGTT |
| *Klebsiella pneumoniae* | Kpn | *phoE* | UF-Kpn-F | **CCATCCAGATTCACTCC**CCTGGATCTGACCCTGCAGTA |
|  |  |  | UR-Kpn-R | CACCTCGACCCACTCACCGTCGCCGTTCTGTTTC |
|  |  |  | AM12C-Kpn-P | CAGGGTAAAAACGAAGGC |
| *Legionella pneumophila* | Lpn | *mip* | UF-Lpn-F | **CCATCCAGATTCACTCC**TGAAGATGAAATTGGTGACTGC |
|  |  |  | UR-Lpn-R | CACCTCGACCCACTCATTCCCAAATCGGCACCAATG |
|  |  |  | AM12C-Lpn-P | CAATGGCTGCAACCGATG |
| *Moraxella catarrhalis* | Mca | *copB* | UF-Mca-F | **CCATCCAGATTCACTCC**CGTGTTGACCGTTTTGACTTT |
|  |  |  | UR-Mca-R | CACCTCGACCCACTCATAGATTAGGTTACCGCTGACG |
|  |  |  | AM12C-Mca-P | CAAAACCGACATCAACCCAA |
| *Mycoplasma pneumoniae* | Mpn | *p1* | UF-Mpn-F | **CCATCCAGATTCACTCC**CTTCTTCAGGCTCAGGTCAA |
|  |  |  | UR-Mpn-R | CACCTCGACCCACTCACGTCACTCGTGCTTGGTAAC |
|  |  |  | AM12C-Mpn-P | GATCTCTCCCCCGTTGAAAAAGT |
| *Mycobacterium tuberculosis* | Mtb | *IS6110* | UF-Mtb-F | **CCATCCAGATTCACTCC**CGACCTACTACGACCACATCAACC |
|  |  |  | UR-Mtb-R | CACCTCGACCCACTCAGTTAGCCACACTTTGCGG |
|  |  |  | AM12C-Mtb-P | ATGGCGAACTCAAGGAGCACAT |
| *Neisseria meningitidis* | Nme | *ctrA* | UF-Nme-F | **CCATCCAGATTCACTCC**CCAAGATCGCCGTTCTGAT |
|  |  |  | UR-Nme-R | CACCTCGACCCACTCAATCTCTGCCTCACTGCCATAA |
|  |  |  | AM12C-Nme-P | GCAGAACGTCAGGATAAATGGAT |
| *Pseudomonas aeruginosa* | Pae | *regA* | UF-Pae-F | **CCATCCAGATTCACTCC**CTGCTGGTGGCACAGGACAT |
|  |  |  | UR-Pae-R | CACCTCGACCCACTCATTGTTGGTGCAGTTCCTCATTG |
|  |  |  | AM12C-Pae-P | CAGATGCTTTGCCTCAA |
| *Staphylococcus aureus* | Sau | *nuc* | UF-Sau-F | **CCATCCAGATTCACTCC**CAAGCCTTGACGAACTAAAGC |
|  |  |  | UR-Sau-R | CACCTCGACCCACTCACTGAAGCAAGTGCATTTACG |
|  |  |  | AM12C-Sau-P | TAAGCCACGTCCATATTTATCAGTT |
| *Stenotrophomonas maltophilia* | Sma | *fdnG* | UF-Sma-F | **CCATCCAGATTCACTCC**CCTGATGTCCGACCTGCTGCC |
|  |  |  | UR-Sma-R | CACCTCGACCCACTCACCCCACCACGAYTTCATCA |
|  |  |  | AM12C-Sma-P | GCCAAGCGCACGCAGAAG |
| *Streptococcus pneumoniae* | Spn | *lytA* | UF-Spn-F | **CCATCCAGATTCACTCC**CAAGGTGCCATGAAGACAGG |
|  |  |  | UR-Spn-R | CACCTCGACCCACTCATTTGATACCATGGCGCCTTCTTT |
|  |  |  | AM12C-Spn-P | GCTGGGTCAAGTACAAGGACACA |
| *Streptococcus pyogenes* | Spy | *dnaseB* | UF-Spy-F | **CCATCCAGATTCACTCC**CCTCAGAGTCAATGCCGTTACAG |
|  |  |  | UR-Spy-R | CACCTCGACCCACTCACCATCACGATTTGCTTCTAACCA |
|  |  |  | AM12C-Spy-P | ACCGAACAAAGAGCTCAAGAATG |
| Influenza A virus | InfA | *M1* | UF-InfA-F | **CCATCCAGATTCACTCC**AAGACCAATCCTGTCACCTCTGA |
|  |  |  | UR-InfA-R | CACCTCGACCCACTCACAAAGCGTCTACGCTGCAGTCC |
|  |  |  | AM12C-InfA-P | CACCGTGCCCAGTGAGCGA |
| Influenza B virus | InfB | *M1* | UF-InfB-F | **CCATCCAGATTCACTCC**GAGACACAATTGCCTACCTGCTT |
|  |  |  | UR-InfB-R | CACCTCGACCCACTCATTCTTTCCCACCGAACCAAC |
|  |  |  | AM12C-InfB-P | AGAAGATGGAGAAGGCAAAGCAGAACTAGC |
| Rhinovirus | Rhino | 5ʹ UTR | UF-Rhino-F | **CCATCCAGATTCACTCC**TGGACAGGGTGTGAAGAGC |
|  |  |  | UR-Rhino-R | CACCTCGACCCACTCACAAAGTAGTCGGTCCCATCC |
|  |  |  | AM12C-Rhino-P | TCCTCCGGCCCCTGAATG |
| Respiratory syncytial virus type A | RSVA | *N* | UF-RSVA-F | **CCATCCAGATTCACTCC**ATGGCTCTTAGCAAAGTCAAGTTG |
|  |  |  | UR-RSVA-R | CACCTCGACCCACTCATGCACATCATAATTRGGAGTRTCA |
|  |  |  | AM12C-RSVA-P | ACACTCAACAAAGATCAACTTCTRTCA |
| Respiratory Syncytial virus type B | RSVB | *N* | UF-RSVB-F | **CCATCCAGATTCACTCC**ATGGCTCTTAGCAAAGTCAAGTTG |
|  |  |  | UR-RSVB-R | CACCTCGACCCACTCATGCACATCATAATTRGGAGTRTCA |
|  |  |  | AM12C-RSVB-P | TAAATAAGGATCAGCTGCTGTCATC |
| Human parainfluenza virus type 1 | HPIV1 | *HN* | UR-HPIV1-F | CACCTCGACCCACTCAGTGATTTAAACCCGGTAATTTCTCA |
|  |  |  | UF-HPIV1-R | **CCATCCAGATTCACTCC**CCTTGTTCCTGCAGCTATTACAGA |
|  |  |  | AM12C-HPIV1-P | GTCGTTGATGTCATAGGT |
| Human parainfluenza virus type 2 | HPIV2 | *HN* | UF-HPIV2-F | **CCATCCAGATTCACTCC**CTGCTTCTGCATGATGCAGC |
|  |  |  | UR-HPIV2-R | CACCTCGACCCACTCATGTTGCTGAGGGGATAAAGC |
|  |  |  | AM12C-HPIV2-P | CATACAATGGGACGCCTA |
| Human parainfluenza virus type 3 | HPIV3 | *HN* | UF-HPIV3-F | **CCATCCAGATTCACTCC**GTCTGGTCTTCCATCTTTGATG |
|  |  |  | UR-HPIV3-R | CACCTCGACCCACTCACCTATATCCTGGCAACCTCG |
|  |  |  | AM12C-HPIV3-P | GCCAACGACTGTTGATGGC |
| Human parainfluenza virus type 4 | HPIV4 | *HN* | UF-HPIV4-F | **CCATCCAGATTCACTCC**CAGAGAGGATCCAGCTGGTG |
|  |  |  | UR-HPIV4-R | CACCTCGACCCACTCAGCAGTATTAGGATAACTTAGTGGCC |
|  |  |  | AM12C-HPIV4-P | TAAAGCTTGCCCAAAAGAATG |
| SARS-CoV-2 | SCV2 | *RdRp/Hel* | UR-SCV2-F | CACCTCGACCCACTCACGCATACAGTCTTRCAGGCT |
|  |  |  | UF-SCV2-R | **CCATCCAGATTCACTCC**GTGTGATGTTGAWATGACATGGTC |
|  |  |  | AMC12-SCV2-P | GTCTACGTATGCAAGCACCACATCTTAA |
| Human specimens | RP | *RNase P* | UF-RP-F | **CCATCCAGATTCACTCC**AGATTTGGACCTGCGAGCG |
|  |  |  | UR-RP-R | CACCTCGACCCACTCAGAGCGGCTGTCTCCACAAGT |
|  |  |  | AM12C-RP-P | TTCTGACCTGAAGGCTCTGCGCG |

*OmpA* (outer membrane protein A gene), *rpoB* (RNA polymerase beta subunit gene), *uidA* (β-D-glucuronidase gene), *hpd* (protein D gene), *cap* (capsulation locus), *phoE* (outer membrane phosphoporin gene), *mip* (macrophage infectivity potentiator gene), *copB* (outer membrane protein gene), *p1* (P1 cytadhesin gene), *IS6110* (insertion sequence 6110), *ctrA* (capsular transporter gene), *regA* (regulatory gene for exotoxin A), *nuc* (nuclease gene), *fdnG* (formate dehydrogenase α subunit gene), *lytA* (autolysin gene), *dnaseB* (DNase B gene), *M1* (matrix protein 1 gene), 5ʹ UTR (5ʹ untranslated region), *N* (nucleoprotein gene), *HN* (hemagglutinin-neuraminidase gene), *RdRP/Hel* (RNA-dependent RNA polymerase and helicase genes).

*^a‍‍^*The 5ʹ end of each probe is labeled with an amine group and a 12-carbon spacer (AM12C) to facilitate efficient coupling to magnetic beads.

*^b‍‍^*The underlined sequences represent unique forward (UF, in bold) and reverse (UR) sequences.

**Table S2.** Reference strains and synthetic oligonucleotides used for analytical performance evaluation

| **Target** | **Probe set** | **Target gene** | **Reference strain/Synthetic oligonucleotide (5ʹ to 3ʹ)** |
| --- | --- | --- | --- |
| *Acinetobacter baumannii* | Aba | *ompA* | *A. baumannii* ATCC 19606 |
| *Bordetella pertussis* | Bpe | *transposase* | *B. pertussis* ATCC 10380 |
| *Chlamydia pneumoniae* | Cpn | *rpoB* | GGATCCGAATATCGTGAGAAATTAGGAGCTCTCTTATTAAATGAGAAAGCACCTGCAGCCAT  TATTCACCGTCGTACAGCAGAAATCGTTGTTCATGAAGGCCTACTCTTTGATCAAGAGACAA  TAGAACGGATAGAACAAGAAGATTTAGTGGATCTTTTAATGCCTAACTGTGAAATGTATGAA  GTGTTGAAAGGACTTCTATCAGATTACGAAACGGCATTACAACGGCTAGAAATCAATTATAA  GACTGAAGTTGAGCATATTCGTGAGGGAGGATCC |
| *E. coli* | Eco | *uidA* | *E. coli* ATCC 43890 |
| *Haemophilus influenzae* | Hin | *hpd* | *H. influenzae* type a ATCC 9006 |
| *Haemophilus influenzae type b* | Hib | *cap* | *H. influenzae* type b ATCC 33533 |
| *Klebsiella pneumoniae* | Kpn | *phoE* | *K. pneumoniae* ATCC 4727 |
| *Legionella pneumophila* | Lpn | *mip* | *L. pneumophila* ATCC 43661 |
| *Moraxella catarrhalis* | Mca | *copB* | *M. catarrhalis* ATCC 25238 |
| *Mycoplasma pneumoniae* | Mpn | *p1* | GGATCCATGAGTGGTAACCTCAGTAGTGTGCTTAGTGGTGGGGGTGCTGGAGGGGGTTCTTC  AGGCTCAGGTCAATCTGGCGTGGATCTCTCCCCCGTTGAAAAAGTGAGTGGGTGGCTTGTGG  GGCAGTTACCAAGCACGAGTGACGGAAACACCTCCTCCACCAACAACCTCGCGGATCC |
| *Mycobacterium tuberculosis* | Mtb | *IS6110* | GAATTCTGTGCCGATCGCCCCATCGACCTACTACGACCACATCAACCGGGAGCCCAGCCGCC  GCGAGCTGCGCGATGGCGAACTCAAGGAGCACATCAGCCGCGTCCACGCCGCCAACTACGGT  GTTTACGGTGCCCGCAAAGTGTGGCTAACCCTGAACCGTGAGGGCATGGATCC |
| *Neisseria meningitidis* | Nme | *ctrA* | *N. meningitidis* ATCC 13090 |
| *Pseudomonas aeruginosa* | Pae | *regA* | *P. aeruginosa* ATCC 10145 |
| *Staphylococcus aureus* | Sau | *N* | *S. aureus* ATCC 25923 |
| *Streptococcus pneumoniae* | Spn | *lytA* | *S. pneumoniae* ATCC 6309 |
| *Streptococcus pyogenes* | Spy | *dnaseB* | *S. pyogenes* ATCC 21547 |
| Influenza A virus | InfA | *M1* | GAATTCCTCATGGAATGGCTAAAGACAAGACCAATCTTGTCACCTCTGACTAAGGGAATTTT  AGGATTTGTGTTCACGCTCACCGTGCCCAGTGAGCGAGGACTGCAGCGTAGACGCTTTGTCC  AAAATGCCCTAAATGGGGGATCC |
| Influenza B virus | InfB | *M1* | GAATTCATGTCGCTGTTTGGAGACACAATTGCCTACCTGCTTTCATTAACAGAAGATGGAGA  AGGCAAAGCAGAACTAGCAGAAAAATTACACTGTTGGTTCGGTGGGAAAGAATTTGACCTAG  ACTCTGCCTTGGATCC |
| Rhinovirus | Rhino | *5ʹ UTR* | GAATTCTGAGGTGTGAAGCCAAGGATTGGACAGGGTGTGAAGAGCCGCGTGTGCTCACTTTG  AGTCCTCCGGCCCCTGAATGCGGCTAACCTTAAACCCGCAGCCATGGCTCATAAACCAATGA  GTTTGTGGTCGTAATGAGTAATTGCGGGATGGGACCGACTACTTTGGGTGTCCGTGTTTCAC  TTTTGGATCC |
| Respiratory syncytial virus type A | RSVA | *N* | GAATTCAAGATGGGGCAAATACAAAAATGGCTCTTAGCAAAGTCAAGTTGAATGATACACTC  AACAAAGATCAACTTCTATCATCCAGCAAATACACCATCCAACGGAGCACAGGAGACAGCAT  TGACACTCCCAATTATGATGTGCAGAAACACATCAATAAGTTATGGATCC |
| Respiratory syncytial virus type B | RSVB | *N* | GAATTCAAATTGGGGCAAATACAAAGATGGCTCTTAGCAAAGTCAAGTTGAATGATACATTA  AATAAGGATCAGCTGCTGTCATCCAGCAAATACACTATTCAACGTAGTACAGGAGATAATAT  TGACACTCCCAATTATGATGTGCAAAAACACCTAAACAAACTATGGATCC |
| Human parainfluenza virus type 1 | HPIV1 | *HN* | GAATTCCTTAAATTCAGATATGTATCCTGATTTAAAACCGGTAATTTCTCATACCTATGACA  TCAACGACAACAGGAAATCATGTTCTGTAATAGCTGCAGGAACAAGGGGTTATCAGTTATGC  TCCTTGGATCC |
| Human parainfluenza virus type 2 | HPIV2 | *HN* | GAATTCCCAACTGTACACCCGGAAATCTGCTTCTGCATGATGCAGCGTACATCAATGGAATA  AACAAATTCCTTGTACTTAAATCATACAATGGGACGCCTAAATATGGACCTCTCCTAAATAT  TCCCAGCTTTATCCCCTCAGCAACATCTCCCAACGGGTGCACTAGGGATCC |
| Human parainfluenza virus type 3 | HPIV3 | *HN* | GAATTCGATGATTTCTGGAGATGCACGTCTGGTCTTCCATCTTTGATGAAAACTCCAAAAAT  AAGATTAATGCCGGGACCAGGATTATTAGCTATGCCAACGACTGTTGATGGCTGTGTCAGAA  CCCCGTCCTTAGTGATAAATGATCTGATTTATGCTTACACCTCAAATCTAATTACTCGAGGT  TGCCAGGATATAGGGAAATCATATCAAGTATTACGGATCC |
| Human parainfluenza virus type 4 | HPIV4 | *HN* | ATTTTATTATCAGAGAGGATCCAGCTGGTGGCCTTATCCGAGCCTCTATAGAATTAGATTGA  ACCTTAGTAATAAATATCCTAAAATAACTGAAATTAAATTTACAAAAATTGAAATCGCCCCA  AGACCAGGTAACAAAGATTGTCCAGGGAATAAAGCTTGCCCAAAAGAATGTATAACAGGAGT  CTATCAAGATACATGGCCACTAAGTTATCCTAATACTGCATTTCCACAC |
| SARS-CoV-2 | SCV2 | *RdRp/Hel* | GAATTCTTATGAGGCTATGTACACACCGCATACAGTCTTACAGGCTGTTGGGGCTTGTGTTC  TTTGCAATTCACAGACTTCATTAAGATGTGGTGCTTGCATACGTAGACCATTCTTATGTTGT  AAATGCTGTTACGACCATGTCATATCAACATCACATAAATTAGTCTTGTCTGTTAAGGATCC |

**Table S3.** Analytical specificity of the PPID assay for respiratory flora and unrelated non-target bacterial species

| Template | **Aba** | **Bpe** | **Cpn** | **Eco** | **Hin** | **Hib** | **Kpn** | **Lpn** | **Mca** | **Mpn** | **Mtb** | **Nme** | **Pae** | **Sau** | **Sma** | **Spn** | **Spy** | **InfA** | **InfB** | **Rhino** | **RSVA** | **RSVB** | **HPIV1** | **HPIV2** | **HPIV3** | **HPIV4** | **SCV2** |
| --- | --- | --- | --- | --- | --- | --- | --- | --- | --- | --- | --- | --- | --- | --- | --- | --- | --- | --- | --- | --- | --- | --- | --- | --- | --- | --- | --- |
| *Haemophilus parainfluenzae* | 0.5 | 0.2 | 0.8 | 0.6 | 0.4 | 0.7 | 0.5 | 1.0 | 0.5 | 1.0 | 1.1 | 0.3 | 0.7 | 0.3 | 1.1 | 0.5 | 1.0 | 1.0 | 1.2 | 0.9 | 0.8 | 1.4 | 1.2 | 1.2 | 0.9 | 0.9 | 1.0 |
|  | 0.5 | 0.2 | 0.8 | 0.7 | 0.8 | 1.0 | 0.4 | 0.9 | 0.8 | 1.0 | 1.0 | 0.2 | 0.6 | 0.3 | 0.9 | 0.9 | 1.0 | 0.9 | 1.3 | 1.1 | 0.8 | 1.4 | 1.2 | 1.1 | 1.1 | 1.1 | 1.0 |
| *Neisseria sicca* | 0.5 | 0.2 | 0.9 | 1.1 | 0.5 | 1.0 | 1.0 | 1.0 | 0.8 | 1.0 | 1.0 | 1.2 | 0.7 | 0.3 | 1.0 | 0.5 | 1.0 | 1.0 | 1.3 | 1.3 | 1.4 | 1.7 | 0.6 | 1.0 | 1.3 | 1.2 | 1.0 |
|  | 0.4 | 0.2 | 0.8 | 1.4 | 0.9 | 0.8 | 1.3 | 0.8 | 0.8 | 1.0 | 1.1 | 1.4 | 0.6 | 0.4 | 1.1 | 0.6 | 1.0 | 1.0 | 1.3 | 1.3 | 1.0 | 1.4 | 0.8 | 1.0 | 1.5 | 1.2 | 1.0 |
| *C. pseudodiph-theriticum* | 0.5 | 0.2 | 0.6 | 1.1 | 1.4 | 1.1 | 0.6 | 1.1 | 0.4 | 1.0 | 1.0 | 0.9 | 0.7 | 0.3 | 0.9 | 0.8 | 1.1 | 0.9 | 1.0 | 1.1 | 1.5 | 1.5 | 0.6 | 1.1 | 1.2 | 1.2 | 1.2 |
|  | 0.5 | 0.2 | 0.7 | 0.9 | 1.5 | 0.9 | 0.5 | 0.8 | 0.6 | 0.8 | 0.9 | 0.5 | 0.6 | 0.7 | 0.9 | 0.8 | 0.9 | 1.0 | 1.0 | 1.0 | 1.3 | 1.1 | 1.0 | 0.9 | 1.1 | 1.3 | 0.9 |
| *Staphylococcus epidermidis* | 0.4 | 0.3 | 1.1 | 0.6 | 1.3 | 0.9 | 1.3 | 1.0 | 1.4 | 1.0 | 1.0 | 0.3 | 0.6 | 0.2 | 1.0 | 0.4 | 0.9 | 0.8 | 1.1 | 1.2 | 0.8 | 1.2 | 1.0 | 1.0 | 1.0 | 1.1 | 1.0 |
|  | 0.4 | 0.2 | 0.7 | 0.8 | 1.3 | 1.1 | 1.4 | 0.9 | 1.3 | 0.9 | 1.0 | 0.4 | 0.7 | 0.3 | 1.0 | 1.3 | 1.0 | 0.9 | 1.1 | 1.0 | 1.0 | 1.3 | 0.9 | 0.9 | 1.0 | 1.0 | 0.9 |
| *Staphylococcus hominis* | 0.5 | 0.5 | 0.7 | 0.5 | 0.3 | 1.0 | 0.3 | 0.9 | 0.6 | 1.0 | 1.0 | 0.2 | 0.8 | 0.3 | 1.1 | 0.4 | 1.0 | 0.9 | 1.2 | 1.1 | 0.7 | 1.1 | 1.0 | 1.0 | 1.0 | 1.0 | 1.0 |
|  | 0.5 | 7.7 | 0.7 | 0.7 | 0.3 | 0.9 | 0.4 | 1.0 | 0.9 | 1.0 | 1.0 | 0.4 | 0.7 | 0.4 | 1.3 | 0.8 | 1.0 | 1.0 | 1.3 | 0.9 | 0.8 | 1.4 | 0.9 | 0.9 | 1.0 | 0.8 | 0.9 |
| *Salmonella typhi* | 0.2 | 1.9 | 0.8 | 0.6 | 0.7 | 1.2 | 1.1 | 1.0 | 1.1 | 1.1 | 1.1 | 2.6 | 0.5 | 0.5 | 1.2 | 1.3 | 1.0 | 1.1 | 1.0 | 0.9 | 1.2 | 1.1 | 0.3 | 1.2 | 1.1 | 1.3 | 1.1 |
|  | 0.2 | 1.8 | 0.8 | 0.3 | 0.8 | 1.1 | 0.9 | 0.9 | **2.2** | 1.1 | 1.0 | 0.7 | 0.4 | 0.6 | 1.0 | 0.5 | 1.0 | 1.3 | 1.7 | 1.1 | 1.3 | 1.0 | 3.0 | 1.3 | 1.2 | 1.3 | 1.2 |
| *Vibrio cholera* | 0.3 | 3.0 | 1.3 | 0.6 | 0.8 | 1.3 | 1.4 | 1.0 | 0.9 | 1.2 | 1.2 | 1.5 | 0.6 | 1.9 | 1.2 | 0.8 | 1.1 | 0.9 | 0.8 | 1.0 | 1.8 | 1.4 | 0.2 | 1.1 | 1.0 | 1.1 | 1.4 |
|  | 0.2 | 0.8 | 1.1 | 0.8 | 0.7 | 1.1 | 1.0 | 1.1 | 0.4 | 1.2 | 1.0 | 0.7 | 0.5 | 0.5 | 1.1 | 0.5 | 1.0 | 1.1 | 1.4 | 1.0 | 1.1 | 1.1 | 0.4 | 1.2 | 1.1 | 1.4 | 1.1 |
| *Vibrio parahaemolyticus* | 0.4 | 0.2 | 0.8 | **8.2** | 0.7 | 1.1 | 1.0 | 0.9 | 0.7 | 1.0 | 1.0 | 0.6 | 0.5 | 0.6 | 1.1 | 1.4 | 1.0 | 1.2 | 1.4 | 1.1 | 1.3 | 0.9 | 0.5 | 1.2 | 1.2 | 1.5 | 1.2 |
|  | 0.2 | 1.4 | 0.7 | 1.5 | 0.6 | 1.1 | 1.0 | 0.9 | 1.0 | 1.1 | 1.0 | 0.6 | 0.5 | 1.0 | 1.1 | 0.6 | 1.0 | 1.0 | 1.2 | 0.9 | 0.9 | 1.1 | 0.3 | 1.1 | 1.9 | 0.9 | 1.1 |
| *Clostridium difficile* | 0.3 | 0.2 | 0.7 | 0.6 | 0.8 | 1.1 | 0.9 | 1.0 | 0.5 | 1.1 | 1.0 | 1.8 | 0.5 | 0.4 | 1.0 | 0.5 | 1.0 | 1.0 | 1.0 | 1.0 | 1.4 | 1.1 | 0.2 | 0.9 | 1.1 | 1.2 | 1.1 |
|  | 0.3 | 1.5 | 0.9 | 0.4 | 1.3 | 1.2 | 1.0 | 0.9 | 1.4 | 1.1 | 1.1 | 0.7 | 0.5 | 0.6 | 1.1 | 0.9 | 1.1 | 1.3 | 1.1 | 1.1 | 1.2 | 1.1 | 5.3 | 1.2 | 1.1 | 1.4 | 1.2 |
| *Clostridium perfringens* | 0.3 | 1.6 | 0.8 | 0.4 | 0.7 | 1.1 | 1.0 | 1.0 | 0.7 | 1.1 | 1.1 | 0.7 | 0.5 | 0.9 | 1.1 | 1.1 | 1.0 | 1.4 | 1.1 | 1.1 | 1.8 | 1.4 | 0.4 | 1.2 | 1.1 | 1.4 | 1.1 |
|  | 0.2 | 0.7 | 0.7 | 0.5 | 0.7 | 1.0 | 1.0 | 0.9 | 0.8 | 1.1 | 1.1 | 1.8 | 0.5 | 0.6 | 1.2 | 0.5 | 1.0 | 1.5 | 1.1 | 1.1 | 1.4 | 1.1 | 3.2 | 1.2 | 1.3 | 1.5 | 1.3 |
| *Campylobacter jejuni* | 0.3 | 0.2 | 0.7 | 1.2 | 0.7 | 1.1 | 1.1 | 1.1 | 0.7 | 1.0 | 1.1 | 3.8 | 0.5 | 0.8 | 1.1 | 0.6 | 1.0 | 1.1 | 1.0 | 0.9 | 0.9 | 0.8 | 3.5 | 1.1 | 0.9 | 1.1 | 1.0 |
|  | 0.3 | 1.0 | 0.8 | 0.8 | 0.8 | 1.2 | 1.0 | 1.0 | 0.6 | 1.2 | 1.1 | 2.7 | 0.5 | 0.5 | 1.2 | 1.6 | 1.0 | 1.2 | 1.1 | 1.1 | 1.3 | 1.5 | 0.3 | 1.4 | 1.2 | 1.2 | 1.3 |
| *Bacillus cereus* | 0.2 | 0.3 | 0.8 | 0.3 | 0.8 | 1.1 | 0.9 | 1.0 | **3.0** | 1.0 | 1.1 | 0.7 | 0.5 | 0.7 | 1.1 | 0.6 | 1.0 | 1.2 | 1.2 | 1.0 | 1.5 | 1.0 | 0.3 | 1.4 | 1.2 | 1.4 | 1.3 |
|  | 0.4 | 2.1 | 0.8 | 0.7 | 1.7 | 1.2 | 1.0 | 0.9 | 0.6 | 1.1 | 1.1 | 1.2 | 1.6 | 0.5 | 0.9 | 1.8 | 1.0 | 1.1 | 0.9 | 1.0 | 1.1 | 0.8 | 0.6 | 1.1 | 1.1 | 1.3 | 1.1 |
| **Positive cutoff** | **2.3** | **8.0** | **2.0** | **2.0** | **5.0** | **2.0** | **2.0** | **3.0** | **2.0** | **3.0** | **2.0** | **5.0** | **2.2** | **2.0** | **2.0** | **2.5** | **2.0** | **10** | **15** | **3** | **5** | **2** | **10** | **2** | **2** | **12** | **5** |

**Table S3 (Continued).** Analytical specificity of the PPID assay for respiratory flora and unrelated non-target bacterial species

Nucleic acids from 5 respiratory flora and 7 unrelated non-target bacterial strains were tested in duplicate at 10⁵ copies/reaction using the PPID assay. Results are expressed as signal-to-noise ratios (SNRs), with values exceeding the positive cutoff shown in bold.

**Table S4.** Precision testing of the PPID assay

| **Template** |  | **Aba** | **Bpe** | **Cpn** | **Eco** | **Hin** | **Hib** | **Kpn** | **Lpn** | **Mca** | **Mpn** | **Mtb** | **Nme** | **Pae** | **Sau** | **Sma** | **Spn** | **Spy** |
| --- | --- | --- | --- | --- | --- | --- | --- | --- | --- | --- | --- | --- | --- | --- | --- | --- | --- | --- |
| **Positive cutoff** |  | **2.3** | **8** | **2** | **2** | **5** | **2** | **2** | **3** | **2** | **3** | **2** | **5** | **2.2** | **2** | **2** | **2.5** | **2** |
| *B. pertussis*  10^5^ copies/reaction | Day 1 | 1.0 | ***33.4*** | 0.9 | 1.5 | 0.8 | 1.0 | 0.8 | 0.9 | 0.4 | **10.9** | 1.0 | 0.1 | 1.0 | 0.6 | 0.8 | 0.6 | 0.7 |
|  |  | 1.2 | ***37.4*** | 1.9 | **2.6** | 0.9 | 1.1 | 0.9 | 1.0 | 0.4 | **11.8** | 0.9 | 0.7 | 1.1 | 1.9 | 0.8 | 0.7 | 0.8 |
|  | Day 2 | 1.2 | ***56.3*** | 1.1 | 1.5 | 0.9 | 1.2 | 0.9 | 1.0 | 0.4 | **16.1** | 1.1 | 0.1 | 1.2 | 1.0 | 1.2 | 0.5 | 0.9 |
|  |  | 0.9 | ***53.5*** | 1.3 | 1.3 | 0.7 | 1.2 | 0.7 | 1.0 | 0.3 | **14.2** | 1.0 | 0.4 | 1.3 | 1.7 | 1.1 | 0.5 | 0.9 |
|  | Day 3 | 1.2 | ***21.9*** | 0.9 | 1.1 | 1.1 | 1.2 | 1.2 | 1.0 | 0.3 | **9.5** | 1.1 | 1.0 | 1.1 | 1.1 | 1.3 | 0.6 | 1.1 |
|  |  | 1.1 | ***21.5*** | 1.0 | 0.8 | 0.9 | 1.1 | 1.0 | 1.0 | 0.2 | **8.8** | 1.1 | 0.7 | 1.1 | 1.0 | 1.2 | 0.8 | 1.1 |
| *B. pertussis* 10^3^ copies/reaction | Day 1 | 0.9 | ***18.4*** | 1.0 | 0.3 | 1.2 | 0.8 | 0.7 | 0.9 | 0.3 | 2.5 | 0.9 | 0.1 | 1.0 | 0.6 | 0.9 | 0.6 | 0.8 |
|  |  | 1.2 | ***28.1*** | 1.1 | 0.3 | 1.5 | 1.4 | 0.8 | 1.0 | 0.3 | 2.7 | 1.0 | 0.2 | 1.1 | 1.0 | 1.1 | 0.7 | 0.9 |
|  | Day 2 | 1.3 | ***8.9*** | 1.0 | 1.2 | 0.8 | 1.0 | 1.7 | 1.0 | 0.2 | 3.0 | 1.0 | 1.5 | 0.9 | 0.9 | 1.0 | 1.1 | 0.8 |
|  |  | 1.3 | ***8.6*** | 0.7 | 0.7 | 1.9 | 1.1 | 1.1 | 1.0 | 0.6 | 2.5 | 1.1 | 0.6 | 1.2 | 1.1 | 1.1 | 0.7 | 1.0 |
|  | Day 3 | 1.3 | ***10.9*** | 1.2 | 1.0 | 0.7 | 1.3 | 0.9 | 0.9 | 0.1 | 2.7 | 1.0 | 1.0 | 1.2 | 1.1 | 1.3 | 0.5 | 0.9 |
|  |  | 1.2 | ***11.1*** | 0.9 | 0.7 | 0.7 | 1.0 | 1.3 | 0.9 | 0.1 | 2.6 | 1.0 | 1.0 | 1.1 | 1.0 | 1.0 | 0.9 | 0.9 |
| *E. coli*  10^5^ copies/reaction | Day 1 | 1.2 | 0.1 | 0.9 | ***94.4*** | 0.9 | 1.3 | 1.2 | 0.9 | 0.1 | 1.0 | 0.9 | 1.6 | 0.9 | 1.2 | 1.3 | 0.8 | 0.8 |
|  |  | 1.1 | 0.1 | 1.0 | ***95.5*** | 0.8 | 1.0 | 1.3 | 0.9 | 0.0 | 1.0 | 0.9 | 1.5 | 0.9 | 0.8 | 1.1 | 0.8 | 0.9 |
|  | Day 2 | 1.4 | 0.2 | 1.1 | ***92.2*** | 0.7 | 1.4 | 1.2 | 1.0 | 0.1 | 1.1 | 1.1 | 1.3 | 1.3 | 1.1 | 1.7 | 0.9 | 1.0 |
|  |  | 1.4 | 0.2 | 1.4 | ***92.4*** | 1.2 | 1.7 | 1.3 | 1.0 | 0.0 | 1.1 | 1.2 | 1.5 | 1.4 | 1.6 | 2.0 | 0.7 | 1.1 |
|  | Day 3 | 1.2 | 0.7 | 0.8 | ***140.9*** | 0.9 | 1.0 | 1.2 | 1.0 | 0.2 | 1.1 | 1.1 | 1.0 | 1.2 | 1.1 | 1.3 | 1.8 | 1.1 |
|  |  | 1.2 | 0.8 | 0.8 | ***140.1*** | 1.0 | 1.2 | 1.3 | 1.0 | 0.2 | 1.1 | 1.1 | 1.6 | 1.1 | 1.2 | 1.3 | 1.7 | 1.1 |
| *E. coli*  10^2^ copies/reaction | Day 1 | 0.8 | 0.1 | 0.9 | ***38.8*** | 1.0 | 0.8 | 0.7 | 0.9 | 0.1 | 1.0 | 0.9 | 0.7 | 0.8 | 0.7 | 0.8 | 0.8 | 0.7 |
|  |  | 1.3 | 0.2 | 1.1 | ***39.9*** | 1.4 | 1.2 | 0.9 | 0.9 | 0.1 | 1.2 | 1.1 | 0.9 | 1.2 | 1.0 | 1.3 | 1.4 | 1.1 |
|  | Day 2 | 1.0 | 3.8 | 1.2 | ***33.2*** | 1.2 | 0.9 | 1.3 | 0.9 | 0.2 | 0.9 | 1.0 | 1.4 | 1.0 | 0.6 | 1.0 | 1.1 | 0.8 |
|  |  | 1.1 | 0.7 | 0.8 | ***35.5*** | 0.9 | 1.1 | 1.1 | 1.0 | 0.2 | 1.1 | 1.0 | 1.0 | 1.1 | 1.1 | 1.1 | 0.6 | 1.0 |
|  | Day 3 | 1.0 | 3.5 | 1.0 | ***41.3*** | 1.2 | 1.0 | 1.0 | 0.9 | 0.3 | 1.0 | 1.0 | 0.6 | 1.1 | 1.1 | 1.0 | 1.0 | 1.0 |
|  |  | 1.1 | 2.7 | 1.0 | ***42.1*** | 1.0 | 1.1 | 0.9 | 1.0 | 0.2 | 1.2 | 1.1 | 0.7 | 1.3 | 1.2 | 1.3 | 0.9 | 1.0 |
| *H. influenzae* type a  10^5^ copies/reaction | Day 1 | 1.1 | 0.1 | 0.9 | 1.0 | ***382.6*** | 1.1 | 1.3 | 0.9 | 0.1 | 1.1 | 0.9 | 1.3 | 1.0 | 0.7 | 0.9 | 0.9 | 0.8 |
|  |  | 1.9 | 0.2 | 1.6 | 1.1 | ***385.0*** | 1.8 | 1.4 | 1.0 | 0.1 | 1.2 | 1.1 | 1.4 | 1.4 | 1.4 | 1.5 | 1.0 | 1.0 |
|  | Day 2 | 1.1 | 4.6 | 1.1 | 1.1 | ***215.4*** | 1.2 | 1.3 | 1.0 | 0.2 | 1.1 | 1.1 | 1.5 | 1.1 | 1.1 | 1.2 | 0.7 | 1.1 |
|  |  | 1.0 | 0.7 | 0.9 | 0.6 | ***213.1*** | 1.2 | 1.0 | 0.9 | 0.1 | 1.1 | 1.0 | 0.6 | 1.0 | 1.1 | 1.2 | 0.5 | 1.0 |
|  | Day 3 | 1.1 | 0.7 | 1.0 | 0.8 | ***205.1*** | 1.2 | 1.3 | 1.0 | 0.2 | 1.2 | 1.1 | 0.7 | 1.2 | 1.2 | 1.2 | 0.7 | 1.1 |
|  |  | 1.0 | 1.4 | 1.0 | 0.8 | ***200.0*** | 1.1 | 1.0 | 1.0 | 0.2 | 1.2 | 1.0 | 0.6 | 1.1 | 1.2 | 1.1 | 0.6 | 1.0 |
| *H. influenzae* type a  10^2^ copies/reaction | Day 1 | 1.0 | 0.1 | 1.4 | 1.0 | ***88.2*** | 1.0 | 1.6 | 0.9 | 0.1 | 1.0 | 0.9 | 1.2 | 1.2 | 1.0 | 0.7 | 1.0 | 0.8 |
|  |  | 1.6 | 0.2 | 1.8 | 1.1 | ***91.6*** | 1.4 | 1.5 | 0.9 | 0.1 | 1.1 | 1.1 | 1.3 | 1.3 | 1.7 | 1.5 | 1.1 | 1.0 |
|  | Day 2 | 1.0 | 0.1 | 1.3 | 0.8 | ***66.9*** | 0.7 | 1.0 | 0.9 | 0.1 | 0.9 | 0.9 | 1.2 | 1.0 | 0.9 | 0.9 | 0.5 | 0.8 |
|  |  | 0.8 | 0.1 | 1.8 | 0.9 | ***66.4*** | 1.0 | 1.3 | 0.9 | 0.3 | 0.9 | 0.9 | 0.1 | 0.8 | 0.7 | 0.9 | 1.2 | 0.7 |
|  | Day 3 | 1.5 | 0.2 | 1.8 | 0.8 | ***82.0*** | 1.7 | 1.1 | 1.0 | 0.1 | 1.1 | 1.1 | 1.2 | 1.3 | 1.5 | 1.5 | 0.5 | 1.0 |
|  |  | 1.2 | 0.2 | 2.5 | 0.7 | ***78.4*** | 1.0 | 1.5 | 0.9 | 0.3 | 0.9 | 1.0 | 0.1 | 1.1 | 1.0 | 1.2 | 1.4 | 0.9 |
| *K. pneumoniae*  10^5^ copies/reaction | Day 1 | 1.0 | 0.1 | 1.1 | 0.3 | 1.1 | 1.1 | ***167.6*** | 1.1 | 0.1 | 1.0 | 1.0 | 0.3 | 1.1 | 0.6 | 0.9 | 0.7 | 0.8 |
|  |  | 1.0 | 0.1 | 1.0 | 0.3 | 0.6 | 1.2 | ***168.7*** | 0.9 | 0.1 | 1.0 | 0.9 | 0.2 | 1.0 | 1.1 | 1.0 | 0.8 | 0.7 |
|  | Day 2 | 1.2 | 1.8 | 0.9 | 1.2 | 1.0 | 1.2 | ***187.8*** | 1.2 | 0.2 | 1.4 | 1.2 | 1.0 | 1.3 | 1.3 | 1.3 | 0.6 | 1.1 |
|  |  | 1.2 | 0.7 | 0.9 | 1.0 | 1.0 | 1.2 | ***182.6*** | 1.0 | 0.4 | 1.1 | 1.1 | 0.9 | 1.2 | 1.1 | 1.3 | 0.6 | 1.0 |
|  | Day 3 | 1.0 | 1.0 | 0.8 | 1.5 | 1.1 | 1.1 | ***180.7*** | 1.0 | 0.2 | 1.1 | 1.0 | 0.9 | 1.3 | 1.1 | 1.2 | 1.2 | 1.1 |
|  |  | 1.1 | 0.9 | 0.9 | 0.8 | 1.2 | 1.2 | ***179.5*** | 1.1 | 0.3 | 1.1 | 1.1 | 0.9 | 1.3 | 1.2 | 1.3 | 1.5 | 1.1 |
| *K. pneumoniae*  10^2^ copies/reaction | Day 1 | 0.9 | 2.2 | 1.0 | 0.3 | 1.3 | 0.7 | ***20.5*** | 0.9 | 0.1 | 0.9 | 1.0 | 0.1 | 1.0 | 0.6 | 0.6 | 1.1 | 0.7 |
|  |  | 0.8 | 1.5 | 0.9 | 0.2 | 0.7 | 0.7 | ***22.1*** | 0.8 | 0.1 | 0.9 | 0.9 | 0.1 | 0.8 | 0.5 | 0.5 | 0.8 | 0.7 |
|  | Day 2 | 1.1 | 0.6 | 1.1 | 0.4 | 1.1 | 1.1 | ***14.6*** | 1.0 | 0.1 | 0.9 | 1.0 | 0.5 | 0.9 | 0.7 | 0.7 | 0.4 | 0.7 |
|  |  | 1.0 | 2.2 | 0.9 | 0.2 | 1.3 | 1.0 | ***13.6*** | 0.8 | 0.1 | 0.9 | 0.9 | 0.1 | 0.9 | 0.8 | 0.7 | 0.9 | 0.7 |
|  | Day 3 | 1.4 | 4.1 | 1.2 | 0.3 | 1.9 | 1.4 | ***9.7*** | 1.0 | 0.1 | 1.1 | 1.1 | 0.2 | 1.3 | 1.2 | 1.5 | 2.5 | 1.0 |
|  |  | 1.0 | 2.5 | 1.0 | 0.2 | 0.8 | 1.1 | ***11.0*** | 0.8 | 0.1 | 1.0 | 1.0 | 0.1 | 1.0 | 0.8 | 1.1 | 1.1 | 0.9 |
| *M. tuberculosis*  10^5^ copies/reaction | Day 1 | 2.0 | 0.3 | 0.7 | 0.3 | 0.7 | 1.0 | 0.6 | 1.0 | **2.2** | 1.0 | ***103.6*** | 0.1 | 1.3 | 0.9 | 0.8 | 0.6 | 0.7 |
|  |  | 2.2 | 0.3 | 1.6 | 1.1 | 1.1 | 2.0 | 1.7 | 1.1 | 1.8 | 1.2 | ***103.8*** | 1.3 | 1.4 | 1.5 | 1.5 | 2.3 | 1.1 |
|  | Day 2 | 1.8 | 0.2 | 1.1 | 0.9 | 0.9 | 1.2 | 1.3 | 1.0 | 1.7 | 1.0 | ***97.4*** | 2.0 | 1.1 | 0.9 | 1.2 | 0.8 | 0.8 |
|  |  | 1.7 | 0.3 | 1.0 | 0.3 | 1.0 | 1.3 | 0.8 | 1.1 | 1.5 | 1.1 | ***86.4*** | 0.1 | 1.6 | 1.2 | 1.4 | 1.1 | 1.0 |
|  | Day 3 | 1.5 | 0.2 | 1.1 | 0.3 | 0.7 | 1.0 | 1.4 | 1.0 | 1.3 | 1.0 | ***75.6*** | 0.4 | 0.9 | **3.1** | 1.1 | 1.1 | 0.8 |
|  |  | 1.6 | 0.2 | 1.2 | 1.0 | 1.1 | 1.8 | 1.5 | 1.0 | 1.3 | 1.2 | ***79.5*** | 1.9 | 1.3 | 1.1 | 1.3 | 0.8 | 1.0 |
| *M. tuberculosis*  5×10^2^ copies/reaction | Day 1 | 2.0 | 0.2 | 1.4 | 0.4 | 1.0 | 1.5 | 1.1 | 1.1 | 0.2 | 1.2 | ***27.2*** | 0.4 | 1.3 | 1.3 | 1.7 | 0.5 | 1.0 |
|  |  | 1.7 | 0.2 | 1.5 | 0.4 | 0.9 | 1.6 | 1.6 | 1.1 | 0.2 | 1.1 | ***25.1*** | 0.2 | 1.3 | 1.7 | 1.6 | 1.1 | 1.0 |
|  | Day 2 | 1.1 | 4.1 | 0.7 | 1.0 | 1.0 | 1.1 | 1.4 | 1.1 | 1.4 | 1.0 | ***25.6*** | 1.4 | 1.1 | 1.1 | 1.1 | 1.0 | 1.0 |
|  |  | 1.3 | 2.7 | 0.8 | 1.1 | 1.1 | 1.3 | 1.2 | 1.1 | 1.5 | 1.1 | ***27.0*** | 1.4 | 1.3 | 1.2 | 1.3 | 0.7 | 1.1 |
|  | Day 3 | 1.1 | 1.0 | 0.8 | 0.9 | 0.9 | 1.2 | 1.2 | 1.1 | 1.5 | 1.2 | ***32.0*** | 0.7 | 1.4 | 1.1 | 1.2 | 1.0 | 1.1 |
|  |  | 1.2 | 4.4 | 0.7 | 0.8 | 1.1 | 1.0 | 1.0 | 1.0 | 1.5 | 1.1 | ***28.3*** | 0.7 | 1.2 | 1.0 | 1.2 | 0.9 | 1.0 |
| *P. aeruginosa*  10^5^ copies/reaction | Day 1 | 1.7 | 3.4 | 1.9 | 0.7 | 1.2 | 1.6 | 1.1 | 1.1 | 0.2 | 1.1 | 1.2 | 0.8 | ***274.6*** | 1.8 | 1.7 | 0.4 | 1.0 |
|  |  | 1.6 | 0.2 | 1.7 | 0.7 | 1.5 | 1.5 | 1.6 | 1.1 | 0.8 | 1.0 | 1.1 | 1.8 | ***264.7*** | 1.0 | 1.2 | 1.1 | 1.0 |
|  | Day 2 | 1.8 | 0.2 | 1.4 | 1.0 | 1.2 | 1.8 | 1.3 | 1.1 | 0.2 | 1.2 | 1.2 | 2.7 | ***244.0*** | 1.4 | 1.7 | 1.2 | 1.1 |
|  |  | 1.7 | 0.5 | 1.4 | 0.3 | 1.3 | 1.6 | 1.2 | 0.9 | 0.3 | 1.1 | 1.1 | 0.2 | ***242.5*** | 1.2 | 1.5 | 1.3 | 1.0 |
|  | Day 3 | 1.2 | 2.6 | 1.0 | 0.8 | 1.1 | 1.1 | 1.1 | 1.0 | 0.3 | 1.0 | 1.1 | 0.7 | ***152.4*** | 1.2 | 1.2 | 0.6 | 1.1 |
|  |  | 1.0 | 3.6 | 1.0 | 0.7 | 1.4 | 1.2 | 1.1 | 1.0 | 0.3 | 1.2 | 1.1 | 0.7 | ***154.2*** | 1.2 | 1.2 | 0.7 | 1.0 |
| *P. aeruginosa*  5×10^2^ copies/reaction | Day 1 | 0.7 | 0.1 | 1.0 | 1.3 | 0.7 | 0.9 | 1.2 | 0.9 | 0.1 | 0.9 | 0.9 | 1.4 | ***45.2*** | 0.5 | 0.8 | 0.7 | 0.8 |
|  |  | 1.1 | 0.7 | 0.8 | 0.7 | 0.9 | 1.0 | 1.1 | 0.9 | 0.2 | 1.1 | 1.1 | 0.6 | ***47.3*** | 1.2 | 1.2 | 0.7 | 1.1 |
|  | Day 2 | 0.8 | 0.1 | 0.8 | 1.0 | 0.7 | 1.0 | 1.0 | 0.9 | 0.1 | 1.0 | 1.0 | 1.1 | ***24.6*** | 0.8 | 0.9 | 0.5 | 1.0 |
|  |  | 1.1 | 3.6 | 0.9 | 0.8 | 1.0 | 1.3 | 1.0 | 1.0 | 0.1 | 1.1 | 1.1 | 0.9 | ***29.0*** | 1.1 | 1.1 | 0.6 | 1.0 |
|  | Day 3 | 1.1 | 3.4 | 0.9 | 0.8 | 1.0 | 1.2 | 1.0 | 1.0 | 0.2 | 1.0 | 1.0 | 0.6 | ***38.8*** | 1.1 | 1.2 | 0.7 | 1.1 |
|  |  | 1.1 | 0.9 | 0.8 | 1.5 | 0.9 | 1.1 | 1.3 | 0.9 | 0.2 | 1.1 | 1.1 | 2.8 | ***31.2*** | 1.1 | 1.2 | 0.7 | 1.1 |
| *S. aureus* 10^5^ copies/reaction | Day 1 | 0.9 | 0.1 | 0.8 | 0.2 | 0.5 | 0.7 | 1.5 | 1.0 | 0.2 | 1.0 | 0.9 | 0.2 | 1.0 | ***229.7*** | 0.8 | 2.0 | 0.8 |
|  |  | 0.9 | 0.4 | 1.1 | 0.5 | 0.6 | 0.9 | 0.6 | 1.0 | 0.1 | 1.0 | 0.9 | 0.9 | 0.9 | ***215.9*** | 0.9 | 0.5 | 0.7 |
|  | Day 2 | 1.0 | 1.5 | 1.2 | 0.5 | 0.6 | 0.8 | 0.9 | 0.9 | 0.2 | 0.9 | 1.0 | 0.4 | 0.9 | ***229.1*** | 0.8 | 0.8 | 0.7 |
|  |  | 1.0 | 0.1 | 0.9 | 0.3 | 0.6 | 1.2 | 1.1 | 0.9 | 0.2 | 1.0 | 1.0 | 0.2 | 0.9 | ***229.7*** | 0.9 | 1.5 | 0.8 |
|  | Day 3 | 1.7 | 1.8 | 1.7 | 0.4 | 1.0 | 1.7 | 1.1 | 1.0 | 0.1 | 1.0 | 1.2 | 0.3 | 1.5 | ***357.9*** | 1.8 | 0.9 | 1.1 |
|  |  | 1.7 | 0.2 | 1.2 | 0.3 | 0.8 | 1.5 | 1.1 | 0.9 | 0.1 | 1.1 | 1.2 | 0.2 | 1.2 | ***355.0*** | 1.5 | 1.5 | 1.0 |
| *S. aureus* 5×10^2^ copies/reaction | Day 1 | 0.9 | 0.1 | 0.9 | 0.4 | 1.2 | 0.9 | 1.1 | 0.9 | 0.3 | 1.0 | 0.9 | 0.2 | 0.9 | ***14.4*** | 1.0 | 0.4 | 0.7 |
|  |  | 1.1 | 4.5 | 1.5 | 1.1 | 1.9 | 1.1 | 1.1 | 1.1 | 0.1 | 1.1 | 1.0 | 1.1 | 1.0 | ***13.9*** | 1.2 | 1.0 | 0.8 |
|  | Day 2 | 0.8 | 0.1 | 0.7 | 1.0 | 0.9 | 1.0 | 1.2 | 0.9 | 0.0 | 1.0 | 0.9 | 1.3 | 0.9 | ***10.3*** | 0.8 | 0.8 | 0.8 |
|  |  | 1.0 | 0.3 | 0.7 | 0.7 | 1.0 | 0.8 | 1.1 | 0.9 | 0.1 | 0.9 | 0.9 | 1.0 | 0.9 | ***9.5*** | 0.7 | 0.5 | 0.8 |
|  | Day 3 | 0.9 | 0.2 | 1.0 | 1.0 | 1.0 | 1.0 | 1.2 | 0.8 | 0.0 | 1.0 | 1.0 | 1.4 | 1.0 | ***4.4*** | 1.0 | 1.0 | 0.9 |
|  |  | 1.2 | 2.0 | 1.1 | 0.4 | 1.0 | 1.4 | 0.8 | 0.9 | 0.1 | 1.0 | 1.0 | 0.4 | 1.1 | ***4.6*** | 1.1 | 0.5 | 0.9 |
| *S. pneumoniae* 10^5^ copies/reaction | Day 1 | 1.0 | 0.1 | 0.7 | 1.3 | 0.8 | 1.0 | 1.8 | 1.0 | 0.1 | 1.0 | 1.0 | 1.3 | 1.0 | 0.8 | 0.9 | ***66.8*** | 0.7 |
|  |  | 1.2 | 0.1 | 0.8 | 1.4 | 0.6 | 0.9 | 1.4 | 1.0 | 0.1 | 1.0 | 1.0 | 1.4 | 0.9 | 0.6 | 0.9 | ***56.1*** | 0.8 |
|  | Day 2 | 1.0 | 0.1 | 0.9 | 1.6 | 0.6 | 0.9 | 1.4 | 1.1 | 0.1 | 1.0 | 1.0 | 1.6 | 0.9 | 0.8 | 0.8 | ***63.7*** | 0.8 |
|  |  | 1.2 | 0.2 | 1.2 | 1.6 | 1.0 | 1.1 | 1.8 | 1.0 | 0.1 | 1.1 | 1.1 | 1.4 | 1.1 | 1.3 | 1.2 | ***66.5*** | 1.1 |
|  | Day 3 | 1.3 | 0.3 | 1.5 | 1.7 | 1.1 | 1.5 | 1.5 | 1.2 | 0.1 | 1.0 | 1.1 | 1.3 | 1.2 | 1.4 | 1.7 | ***55.2*** | 1.0 |
|  |  | 1.4 | 0.2 | 1.1 | 1.4 | 0.8 | 1.3 | 1.4 | 1.1 | 0.0 | 1.1 | 1.2 | 1.3 | 1.3 | 1.3 | 1.3 | ***68.3*** | 1.0 |
| *S. pneumoniae* 5×10^2^ copies/reaction | Day 1 | 1.7 | 0.4 | 1.2 | 1.7 | 1.1 | 1.6 | 1.2 | 1.0 | 0.1 | 1.2 | 1.2 | 0.8 | 1.3 | 1.3 | 1.6 | ***36.7*** | 1.0 |
|  |  | 1.8 | 0.4 | 1.2 | 1.2 | 1.6 | 1.5 | 2.0 | 0.9 | 0.1 | 1.1 | 1.2 | 1.4 | 1.2 | 1.3 | 1.6 | ***33.2*** | 1.1 |
|  | Day 2 | 0.9 | 0.7 | 0.7 | 0.5 | 1.1 | 1.1 | 1.0 | 1.1 | 0.6 | 0.9 | 1.0 | 0.6 | 1.0 | 1.6 | 1.1 | ***26.3*** | 1.0 |
|  |  | 1.2 | 0.6 | 0.9 | 1.8 | 1.0 | 1.0 | 1.1 | 1.0 | 0.6 | 1.1 | 1.0 | 1.9 | 1.0 | 1.1 | 1.1 | ***28.3*** | 1.0 |
|  | Day 3 | 1.0 | 0.8 | 0.9 | 1.1 | 1.0 | 1.0 | 1.0 | 0.9 | 0.2 | 1.1 | 1.0 | 0.7 | 1.1 | 1.2 | 1.2 | ***28.6*** | 1.1 |
|  |  | 1.0 | 0.7 | 0.9 | 1.0 | 0.9 | 1.1 | 1.1 | 1.0 | 1.1 | 1.2 | 1.1 | 0.7 | 1.2 | 1.2 | 1.2 | ***29.5*** | 1.1 |

**Table S4 (Continued).** Precision testing of the PPID assay

Nucleic acids of bacterial targets at moderate-to-high and low concentrations were tested in duplicate for each concentration on three separate days to evaluate intra- and inter-assay variability. Results are expressed as SNRs. SNRs exceeding the predefined cutoff values are shown in bold. Values corresponding to the specific probe set for each nucleic acid template are shown in italics.

**Table S5.** Interference testing for the PPID Assay

| **Specimen No. (spiked materials)** | **Aba** | **Bpe** | **Cpn** | **Eco** | **Hin** | **Hib** | **Kpn** | **Lpn** | **Mca** | **Mpn** | **Mtb** | **Nme** | **Pae** | **Sau** | **Sma** | **Spn** | **Result** |
| --- | --- | --- | --- | --- | --- | --- | --- | --- | --- | --- | --- | --- | --- | --- | --- | --- | --- |
| #025 (None) | 1.0 | 0.9 | 0.8 | 2.1 | ***22.5*** | 1.6 | 1.1 | 0.9 | 0.4 | 1.1 | 1.1 | 3.6 | 1.1 | 0.7 | 1.2 | 1.0 | *H. influenzae* |
|  | 1.4 | 0.8 | 1.0 | 1.4 | ***26.9*** | 1.5 | 1.1 | 1.1 | 0.8 | 1.2 | 1.2 | 4.1 | 1.0 | 1.1 | 1.6 | 1.0 |  |
| #025 (78 μM Hemoglobin) | 1.1 | 0.6 | 0.6 | 1.4 | 1.3 | 1.3 | 0.6 | 0.9 | 0.3 | 1.1 | 1.1 | 3.2 | 1.1 | 0.6 | 1.1 | 1.0 | ND |
|  | 1.4 | 0.8 | 0.7 | 1.3 | 1.4 | 1.4 | 0.7 | 1.0 | 0.4 | 1.1 | 1.2 | 3.3 | 1.1 | 0.7 | 1.1 | 1.1 |  |
| #025 (155 μM Hemoglobin) | 1.5 | 0.8 | 0.8 | 1.1 | 0.9 | 1.6 | 0.7 | 1.1 | 0.4 | 1.1 | 1.2 | 2.7 | 1.1 | 0.9 | 1.2 | 1.1 | ND |
|  | 1.6 | 0.7 | 0.7 | 1.1 | 0.8 | 1.5 | 0.6 | 1.0 | 0.3 | 1.0 | 1.1 | 2.8 | 1.1 | 0.7 | 1.3 | 1.2 |  |
| #025 (10% Nasal spray) | 1.4 | 0.8 | 1.1 | 1.4 | ***41.0*** | 1.7 | 1.1 | 1.3 | 0.5 | 1.1 | 1.1 | 3.4 | 1.1 | 0.9 | 1.2 | 1.0 | *H. influenzae* |
|  | 1.1 | 0.4 | 0.7 | 1.4 | ***38.8*** | 1.5 | 1.0 | 1.0 | 0.4 | 0.9 | 0.9 | 2.2 | 0.9 | 0.6 | 0.8 | 1.0 |  |
| #025 (15% Nasal spray) | 1.6 | 0.5 | 1.3 | 1.4 | ***31.4*** | 1.3 | 0.6 | 1.2 | 0.7 | 1.0 | 1.1 | 1.5 | 1.1 | 1.8 | 1.2 | 1.0 | *H. influenzae* |
|  | 1.3 | 0.7 | 1.3 | 1.9 | ***34.5*** | 1.2 | 1.0 | 1.5 | 0.7 | 1.1 | 1.0 | 4.6 | 0.9 | 0.6 | 1.2 | 0.8 |  |
| #029 (None) | 1.0 | 0.5 | 0.4 | 0.4 | 0.6 | 1.0 | ***68.0*** | 0.9 | 0.4 | 0.9 | 1.0 | 0.6 | 0.8 | 0.7 | 0.9 | 0.9 | *K. pneumoniae* |
|  | 1.5 | 0.8 | 0.7 | 0.3 | 0.8 | 1.5 | ***61.1*** | 1.0 | 0.5 | 1.1 | 1.1 | 0.5 | 1.0 | 1.0 | 1.3 | 1.0 |  |
| #029 (78 μM Hemoglobin) | 0.9 | 1.9 | 0.5 | 0.3 | 0.7 | 1.2 | ***8.6*** | 1.0 | 0.4 | 1.0 | 0.9 | 0.3 | 0.9 | 0.6 | 1.1 | 0.9 | *K. pneumoniae* |
|  | 1.3 | 2.3 | 0.6 | 0.6 | 0.7 | 1.6 | ***7.8*** | 0.9 | 0.4 | 1.0 | 1.1 | 0.5 | 0.9 | 0.9 | 1.3 | 1.0 |  |
| #029 (155 μM Hemoglobin) | 1.2 | 0.7 | 0.7 | 1.2 | 0.7 | 1.3 | 0.7 | 1.1 | 0.4 | 1.0 | 1.1 | 2.9 | 0.9 | 0.7 | 1.0 | 1.0 | ND |
|  | 1.2 | 0.6 | 0.7 | 1.1 | 0.9 | 1.3 | 0.6 | 1.0 | 0.3 | 1.0 | 1.1 | 2.9 | 0.9 | 0.5 | 1.0 | 1.1 |  |
| #029 (10% Nasal spray) | 1.2 | 0.8 | 0.7 | 0.6 | 0.6 | 1.2 | ***37.3*** | 0.9 | 0.4 | 0.9 | 1.2 | 0.5 | 1.0 | 0.6 | 1.2 | 0.9 | *K. pneumoniae* |
|  | 1.5 | 0.7 | 0.5 | 0.3 | 0.8 | 1.5 | ***48.6*** | 1.0 | 0.4 | 1.0 | 1.1 | 0.4 | 1.0 | 1.0 | 1.1 | 1.0 |  |
| #029 (15% Nasal spray) | 0.9 | 0.8 | 0.5 | 0.3 | 0.6 | 1.1 | ***27.2*** | 0.9 | 0.3 | 1.0 | 1.0 | 0.3 | 0.8 | 0.6 | 1.1 | 1.0 | *K. pneumoniae* |
|  | 1.0 | 0.6 | 0.7 | 0.3 | 0.7 | 1.3 | ***36.4*** | 0.9 | 0.3 | 1.1 | 1.1 | 0.3 | 0.8 | 1.0 | 1.3 | 0.9 |  |
| #031 (None) | 1.3 | 0.6 | 0.8 | 1.1 | 0.8 | 1.5 | ***48.9*** | 1.1 | 0.3 | 1.2 | 1.1 | 0.7 | 1.1 | 1.1 | 1.1 | 1.0 | *K. pneumoniae* |
|  | 1.0 | 0.7 | 0.7 | 0.5 | 1.0 | 1.5 | ***47.8*** | 0.9 | 0.5 | 1.1 | 0.9 | 0.5 | 1.0 | 1.1 | 1.1 | 1.0 |  |
| #031 (78 μM Hemoglobin) | 0.6 | 2.4 | 0.5 | 0.4 | 0.7 | 1.4 | ***8.1*** | 0.9 | 0.2 | 0.9 | 1.0 | 0.5 | 0.8 | 0.6 | 1.1 | 0.8 | *K. pneumoniae* |
|  | 1.1 | 2.4 | 0.7 | 2.0 | 0.7 | 1.4 | ***7.2*** | 0.8 | 0.4 | 0.9 | 0.9 | 0.4 | 0.8 | 0.7 | 1.0 | 0.8 |  |
| #031 (155 μM Hemoglobin) | 1.1 | 0.7 | 0.7 | 1.1 | 0.6 | 1.3 | 0.6 | 1.0 | 0.3 | 1.0 | 1.1 | 2.7 | 0.9 | 0.6 | 1.0 | 1.0 | ND |
|  | 1.1 | 0.7 | 0.7 | 1.1 | 0.8 | 1.5 | 0.6 | 0.9 | 0.3 | 0.9 | 1.1 | 3.0 | 1.0 | 0.7 | 1.2 | 1.0 |  |
| #031 (10% Nasal spray) | 1.0 | 0.8 | 1.1 | 0.9 | 0.7 | 1.3 | ***78.4*** | 1.6 | 1.1 | 3.6 | 1.0 | 2.6 | 1.1 | 1.0 | 1.2 | 0.9 | *K. pneumoniae* |
|  | 1.5 | 0.7 | 1.2 | 1.3 | 1.0 | 1.4 | ***48.1*** | 1.4 | 0.5 | 1.1 | 1.2 | 1.3 | 1.0 | 0.8 | 1.1 | 0.9 |  |
| #031 (15% Nasal spray) | 0.9 | 0.8 | 1.3 | 1.0 | 0.7 | 1.7 | ***32.0*** | 1.4 | 0.6 | 1.1 | 1.1 | 1.9 | 1.0 | 0.6 | 1.3 | 0.9 | *K. pneumoniae* |
|  | 1.3 | 0.8 | 1.4 | 2.2 | 0.8 | 1.5 | ***30.4*** | 1.2 | 0.7 | 1.1 | 1.2 | 1.5 | 0.9 | 0.6 | 1.0 | 1.0 |  |
| #033 (None) | 0.9 | 0.5 | 0.6 | 0.8 | 0.7 | 1.0 | 0.8 | 0.9 | 0.8 | 0.8 | 1.0 | 1.8 | ***4.1*** | 0.7 | 1.6 | 0.9 | *P. aeruginosa* |
|  | 1.1 | 0.6 | 0.8 | 1.5 | 0.6 | 1.2 | 0.7 | 1.1 | 1.4 | 1.0 | 1.0 | 1.0 | ***5.1*** | 0.8 | 1.6 | 0.9 |  |
| #033 (78 μM Hemoglobin) | 1.0 | 0.6 | 0.6 | 1.1 | 0.9 | 1.1 | 0.6 | 0.9 | 0.3 | 1.0 | 1.1 | 2.8 | ***7.0*** | 0.6 | 1.1 | 1.0 | *P. aeruginosa* |
|  | 1.0 | 0.5 | 0.5 | 1.1 | 1.0 | 1.3 | 0.5 | 0.8 | 0.3 | 0.9 | 0.9 | 2.7 | ***6.8*** | 0.5 | 0.7 | 0.9 |  |
| #033 (155 μM Hemoglobin) | 0.9 | 0.6 | 0.5 | 1.1 | 0.7 | 1.1 | 0.7 | 0.8 | 0.3 | 1.0 | 1.0 | 2.7 | 1.7 | 0.6 | 0.9 | 0.9 | ND |
|  | 1.1 | 0.7 | 0.5 | 1.0 | 0.7 | 1.2 | 0.7 | 1.0 | 0.3 | 1.1 | 1.0 | 2.9 | 2.1 | 0.6 | 1.1 | 1.0 |  |
| #033 (10% Nasal spray) | 1.6 | 0.8 | 0.7 | 1.8 | 0.8 | 1.4 | 1.1 | 1.0 | 0.6 | 1.0 | 1.0 | 1.7 | ***4.6*** | 0.9 | 1.6 | 1.0 | *P. aeruginosa* |
|  | 1.4 | 0.5 | 0.6 | 1.3 | 1.0 | 1.1 | 1.0 | 0.9 | 0.3 | 1.0 | 1.0 | 3.8 | ***4.5*** | 0.7 | 1.8 | 1.0 |  |
| #033 (15% Nasal spray) | 1.1 | 0.5 | 0.9 | 1.4 | 0.7 | 1.4 | 1.9 | 1.1 | 0.6 | 1.0 | 1.0 | 4.4 | ***6.4*** | 0.8 | 1.9 | 1.0 | *P. aeruginosa* |
|  | 1.3 | 0.6 | 1.0 | 1.6 | 0.5 | 1.1 | 0.9 | 1.0 | 0.3 | 1.0 | 1.0 | 3.2 | ***5.0*** | 0.7 | 1.9 | 0.9 |  |
| **Positive cutoff** | **2.3** | **8.0** | **2.0** | **2.0** | **5.0** | **2.0** | **2.0** | **3.0** | **2.0** | **3.0** | **2.0** | **5.0** | **2.2** | **2.0** | **2.0** | **2.5** |  |

Results are expressed as SNRs, with values exceeding the positive cutoff shown in bold and italic.

**Table S6.** Pathogen identification in clinical specimens using the PPID assay and MC methods (N = 135)

| **Specimen** | **Types of Pneumonia** | **Comorbidity** | **PPID** | |  | **MC** | | **Agreement Category*^c^*** |
| --- | --- | --- | --- | --- | --- | --- | --- | --- |
|  |  |  | **Bacteria*‍‍^a‍,‍b,d^*** | **Virus‍‍*^a,d^*** |  | **PPID target pathogens**‍‍*^b^* | **Non-PPID target pathogens‍‍*^a^*** |  |
| 001 | CAP | Cardiovascular (CAD); Cardiovascular (Heart Failure); Endocrine (Diabetes) | ND | ND |  | No growth |  | Full agreement |
| 002 | CAP | Cardiovascular (Hypertension); Endocrine (Diabetes) | ND | ND |  | No growth |  | Full agreement |
| 003 | CAP | Cardiovascular (Hypertension); Endocrine (Diabetes) | ND | ND |  | No growth |  | Full agreement |
| 004 | CAP | Cardiovascular (Heart Failure); Cardiovascular (Hypertension); Endocrine (Diabetes); Respiratory (COPD) | ND | ND |  | No growth |  | Full agreement |
| 005 | CAP | Cardiovascular (Heart Failure); Cardiovascular (Hypertension) | ND | ND |  | No growth |  | Full agreement |
| 006 | CAP | No Comorbidity | ND | ND |  | No growth |  | Full agreement |
| 007 | CAP | Cancer | ND | ND |  | No growth |  | Full agreement |
| 008 | CAP | Cardiovascular (Hypertension); Endocrine (Diabetes) | ND | ND |  | Mixed flora |  | Full agreement |
| 009 | CAP | Cardiovascular (CAD); Cardiovascular (Heart Failure); Endocrine (Diabetes); Other/Uncategorized | ND | ND |  | Mixed flora |  | Full agreement |
| 010 | CAP | Cancer | ND | ND |  | Normal pharyngeal flora |  | Full agreement |
| 011 | CAP | Cancer; Cardiovascular (Hypertension) | ND | ND |  | Normal pharyngeal flora |  | Full agreement |
| 012 | CAP | Cancer; Cardiovascular (CAD); Cardiovascular (Hypertension); Respiratory (COPD) | ND | ND |  | Normal pharyngeal flora |  | Full agreement |
| 013 | CAP | Cardiovascular (Heart Failure); Hepatic (Liver Cirrhosis) | ND | ND |  | Normal pharyngeal flora |  | Full agreement |
| 014 | CAP | Cancer; Cardiovascular (CAD); Cardiovascular (Hypertension) | ND | ND |  | Normal pharyngeal flora |  | Full agreement |
| 015 | HAP | Cancer | ND | ND |  | No growth |  | Full agreement |
| 016 | HAP | Cardiovascular (Heart Failure); Respiratory (COPD) | ND | ND |  | Normal pharyngeal flora |  | Full agreement |
| 017 | HAP | Cancer; Cardiovascular (Hypertension); Cardiovascular (Heart Failure); Endocrine (Diabetes); Respiratory (COPD) | ND | ND |  | Normal pharyngeal flora |  | Full agreement |
| 018 | HCAP | Cardiovascular (Hypertension); Endocrine (Diabetes) | ND | ND |  | No growth |  | Full agreement |
| 019 | HCAP | Cardiovascular (CAD); Cardiovascular (Heart Failure); Cardiovascular (Hypertension) | ND | ND |  | Normal pharyngeal flora |  | Full agreement |
| 020 | CAP | No Comorbidity | ***A. baumannii*** | ND |  | ***A. baumannii*** |  | Full agreement |
| 021 | CAP | Cardiovascular (Hypertension) | ***K. pneumoniae*** | ND |  | ***K. pneumoniae*** |  | Full agreement |
| 022 | CAP | No Comorbidity | ***K. pneumoniae*** | ND |  | ***K. pneumoniae*** |  | Full agreement |
| 023 | CAP | Cardiovascular (Heart Failure) | ***M. catarrhalis*** | ND |  | ***M. catarrhalis*** |  | Full agreement |
| 024 | CAP | Respiratory (COPD) | ***K. pneumoniae*** | ND |  | ***K. pneumoniae*** |  | Full agreement |
| 025 | CAP | No Comorbidity | ***H. influenzae*** | ND |  | ***H. influenzae*** |  | Full agreement |
| 026 | CAP | Respiratory (COPD) | ***H. influenzae*** | ND |  | ***H. influenzae*** |  | Full agreement |
| 027 | CAP | Cardiovascular (Hypertension) | ***K. pneumoniae*** | ND |  | ***K. pneumoniae*** |  | Full agreement |
| 028 | HCAP | Cardiovascular (CAD); Cardiovascular (Heart Failure); Cardiovascular (Hypertension) | ***S. pneumoniae*** | ND |  | ***S. pneumoniae*** | *Proteus mirabilis Streptococcus agalactiae* | Full agreement |
| 029 | CAP | No Comorbidity | ***K. pneumoniae*** | ND |  | ***K. pneumoniae*** | *Candida. albicans* | Full agreement |
| 030 | CAP | Cancer | ***K. pneumoniae*** | ND |  | ***K. pneumoniae*** | *C. albicans* | Full agreement |
| 031 | CAP | Cardiovascular (Hypertension); Endocrine (Diabetes) | ***K. pneumoniae*** | ND |  | ***K. pneumoniae*** | *C. albicans* | Full agreement |
| 032 | CAP | Cardiovascular (CAD); Cardiovascular (Hypertension) | ***E. coli*** | ND |  | ***E. coli*** | *C. albicans* Yeast Not *C. albicans* | Full agreement |
| 033 | HAP | Cardiovascular (Hypertension); Cardiovascular (Heart Failure) | ***P. aeruginosa*** | ND |  | ***P. aeruginosa*** | *Elizabethkingia anophelis Providencia stuartii* | Full agreement |
| 034 | HAP | Cardiovascular (CAD); Cardiovascular (Heart Failure); Endocrine (Diabetes) | ***P. aeruginosa*** | ND |  | ***P. aeruginosa*** | *E. anophelis Serratia marcescens* | Full agreement |
| 035 | HCAP | Cardiovascular (Hypertension) | ***K. pneumoniae****, E. coli* | ND |  | ***K. pneumoniae*** |  | Partial agreement (PPID > MC) |
| 036 | HCAP | Cardiovascular (Heart Failure) | ***K. pneumoniae****, M. catarrhalis* | ND |  | ***K. pneumoniae*** |  | Partial agreement (PPID > MC) |
| 037 | CAP | Cardiovascular (CAD); Cardiovascular (Hypertension) | ***K. pneumoniae****, H. influenzae* | ND |  | ***K. pneumoniae*** |  | Partial agreement (PPID > MC) |
| 038 | CAP | Cardiovascular (Heart Failure); Cardiovascular (Hypertension) | ***K. pneumoniae****, E. coli,*  *M. catarrhalis* | ND |  | ***K. pneumoniae*** |  | Partial agreement (PPID > MC) |
| 039 | CAP | No Comorbidity | ***E. coli****,*  *K. pneumoniae, P. aeruginosa* | ND |  | ***E. coli*** | *P. stuartii* | Partial agreement (PPID > MC) |
| 040 | HAP | Cardiovascular (Heart Failure); Cardiovascular (Hypertension) | ***S. maltophilia****, H. influenzae,*  *P. aeruginosa* | ND |  | ***S. maltophilia*** | *S. marcescens* | Partial agreement (PPID > MC) |
| 041 | CAP | No Comorbidity | ***S. aureus****,*  *E. coli,*  *K. pneumoniae* | ND |  | ***S. aureus*** |  | Partial agreement (PPID > MC) |
| 042 | CAP | No Comorbidity | ***K. pneumoniae****, A. baumannii,*  *H. influenzae* | ND |  | ***K. pneumoniae*** | *S. agalactiae* | Partial agreement (PPID > MC) |
| 043 | HCAP | Cardiovascular (Hypertension) | ***S. maltophilia****, A. baumannii,*  *P. aeruginosa* | ND |  | ***S. maltophilia*** |  | Partial agreement (PPID > MC) |
| 044 | HAP | Cardiovascular (Hypertension) | ***A. baumannii****,*  *S. aureus,*  *S. maltophilia* | ND |  | ***A. baumannii*** |  | Partial agreement (PPID > MC) |
| 045 | HAP | Cardiovascular (Hypertension); Endocrine (Diabetes) | ***A. baumannii****, E. coli,*  *K. pneumoniae, S. aureus* | ND |  | ***A. baumannii*** | *Elizabethkingia meningoseptica* | Partial agreement (PPID > MC) |
| 046 | CAP | No Comorbidity | ***K. pneumoniae****, A. baumannii,*  *P. aeruginosa,*  *S. pneumoniae* | ND |  | ***K. pneumoniae*** |  | Partial agreement (PPID > MC) |
| 047 | HAP | Cardiovascular (CAD); Endocrine (Diabetes) | ***P. aeruginosa****,* ***S. maltophilia****, K. pneumoniae* | ND |  | ***P. aeruginosa S. maltophilia*** | Non tuberculosis Mycobacterium | Partial agreement (PPID > MC) |
| 048 | CAP | No Comorbidity | ***K. pneumoniae****,* ***S. aureus****,*  *E. coli,*  *P. aeruginosa* | ND |  | ***K. pneumoniae S. aureus*** | *Cupriavidus pauculus* Yeast not Candida | Partial agreement (PPID > MC) |
| 049 | CAP | Cardiovascular (CAD); Cardiovascular (Hypertension); Endocrine (Diabetes); | ***S. aureus*** | HPIV-3 |  | ***S. aureus*** |  | Full agreement |
| 050 | CAP | Endocrine (Diabetes) | ***K. pneumoniae****, H. influenzae* | InfA |  | ***K. pneumoniae*** |  | Partial agreement (PPID > MC) |
| 051 | CAP | Cancer | ***P. aeruginosa****, A. baumannii,*  *K. pneumoniae* | InfA |  | ***P. aeruginosa*** |  | Partial agreement (PPID > MC) |
| 052 | CAP | Cardiovascular (Hypertension) | ***A. baumannii*** | ND |  | ***A. baumannii*** *S. aureus* | *Enterobacter cloacae* | Partial agreement (MC > PPID) |
| 053 | CAP | Cardiovascular (Hypertension); Endocrine (Diabetes) | ***P. aeruginosa****,*  *S. pneumoniae* | ND |  | ***P. aeruginosa*** *A. baumannii* | *C. albicans* | Partial agreement (PPID ≸ MC) |
| 054 | CAP | Cancer; Cardiovascular (CAD); Cardiovascular (Heart Failure); Cardiovascular (Hypertension); Endocrine (Diabetes) | ***E. coli****,*  *P. aeruginosa* | ND |  | ***E. coli***  *S. maltophilia* |  | Partial agreement (PPID ≸ MC) |
| 055 | HAP | Cancer; Cardiovascular (Hypertension); Respiratory (COPD) | *E. coli,*  *K. pneumoniae* | ND |  | *A. baumannii* |  | No agreement (PPID ≸ MC) |
| 056 | CAP | Cardiovascular (Hypertension) | *E. coli* | ND |  | *A. baumannii* |  | No agreement (PPID ≸ MC) |
| 057 | HAP | No Comorbidity | *A. baumannii* | ND |  | *P. aeruginosa* |  | No agreement (PPID ≸ MC) |
| 058 | HCAP | No Comorbidity | *A. baumannii,*  *P. aeruginosa* | ND |  | *S. maltophilia* | *Klebsiella oxytoca S. marcescens* | No agreement (PPID ≸ MC) |
| 059 | CAP | Cardiovascular (Hypertension); Respiratory (COPD) | *A. baumannii,*  *K. pneumoniae, P. aeruginosa* | ND |  | *S. aureus* | *C. albicans* | No agreement (PPID ≸ MC) |
| 060 | HAP | Cardiovascular (Hypertension); Respiratory (COPD) | *A. baumannii,*  *K. pneumoniae* | ND |  | *S. maltophilia* |  | No agreement (PPID ≸ MC) |
| 061 | CAP | Cardiovascular (CAD); Endocrine (Diabetes) | *K. pneumoniae* | ND |  |  | Yeast | No agreement (PPID > MC) |
| 062 | HAP | Cancer; Respiratory (COPD) | *K. pneumoniae, S. maltophilia* | ND |  |  | *E. cloacae* | No agreement (PPID > MC) |
| 063 | CAP | Cardiovascular (Heart Failure); Cardiovascular (Hypertension) | *E. coli,*  *K. pneumoniae* | ND |  |  | Yeast Not *Candida albicans* | No agreement (PPID > MC) |
| 064 | CAP | Cardiovascular (Hypertension) | *K. pneumoniae, S. aureus* | ND |  |  | *C. albicans* | No agreement (PPID > MC) |
| 065 | CAP | No Comorbidity | *H. influenzae,*  *P. aeruginosa* | ND |  |  | *Morganella morganii* | No agreement (PPID > MC) |
| 066 | CAP | Respiratory (COPD) | *A. baumannii,*  *E. coli,*  *H. influenzae,*  *P. aeruginosa* | ND |  |  | *S. agalactiae* | No agreement (PPID > MC) |
| 067 | CAP | Cancer; Cardiovascular (Hypertension) | ND | RSV-B |  |  | *C. albicans* | Full agreement |
| 068 | HCAP | Cardiovascular (Heart Failure); Respiratory (COPD); Cancer | ND | Rhino |  |  | *C. albicans* | Full agreement |
| 069 | HCAP | Cardiovascular (Heart Failure); Cardiovascular (Hypertension) | ND | InfA |  | *P. aeruginosa* |  | No agreement (MC > PPID) |
| 070 | CAP | Cancer | ND | ND |  | *S. aureus* |  | No agreement (MC > PPID) |
| 071 | CAP | Cancer; Cardiovascular (CAD); Cancer | ND | ND |  | *H. influenzae* | Beta-Streptococcus Non-A,B,D. | No agreement (MC > PPID) |
| 072 | CAP | No Comorbidity | ND | ND |  | *A. baumannii* |  | No agreement (MC > PPID) |
| 073 | CAP | Cardiovascular (Heart Failure) | ND | ND |  | *M. catarrhalis* |  | No agreement (MC > PPID) |
| 074 | HAP | Cardiovascular (CAD); Cardiovascular (Heart Failure); Cardiovascular (Hypertension); Endocrine (Diabetes) | ND | ND |  | *S. maltophilia* | Yeast Not *Candida albicans* | No agreement (MC > PPID) |
| 075 | HCAP | Cardiovascular (Hypertension) | ND | ND |  | *H. influenzae* | Beta-Streptococcus Non-A,B,D. *S. agalactiae* | No agreement (MC > PPID) |
| 076 | CAP | Cancer | ND | ND |  |  | *C. albicans* | Full agreement |
| 077 | CAP | Respiratory (COPD) | ND | ND |  |  | *C. albicans* | Full agreement |
| 078 | CAP | Cardiovascular (Hypertension); Endocrine (Diabetes) | ND | ND |  |  | *C. albicans* | Full agreement |
| 079 | CAP | No Comorbidity | ND | ND |  |  | *C. albicans* | Full agreement |
| 080 | HCAP | Endocrine (Diabetes); Respiratory (COPD) | ND | ND |  |  | *C. albicans* | Full agreement |
| 081 | CAP | Cardiovascular (CAD); Cardiovascular (Hypertension); Endocrine (Diabetes) | *H. influenzae* | ND |  | No growth |  | No agreement (PPID > MC) |
| 082 | HAP | Cardiovascular (Hypertension) | *H. influenzae* | ND |  | No growth |  | No agreement (PPID > MC) |
| 083 | CAP | Cardiovascular (CAD); Cardiovascular (Heart Failure); Respiratory (COPD) | *H. influenzae* | ND |  | No growth |  | No agreement (PPID > MC) |
| 084 | CAP | Cancer; Cardiovascular (Hypertension); Endocrine (Diabetes); Respiratory (COPD) | *H. influenzae* | ND |  | No growth |  | No agreement (PPID > MC) |
| 085 | HCAP | Cancer | *K. pneumoniae* | ND |  | No growth |  | No agreement (PPID > MC) |
| 086 | CAP | Cardiovascular (Heart Failure); Cardiovascular (Hypertension) | *K. pneumoniae* | ND |  | No growth |  | No agreement (PPID > MC) |
| 087 | CAP | No Comorbidity | *K. pneumoniae* | ND |  | No growth |  | No agreement (PPID > MC) |
| 088 | CAP | No Comorbidity | *K. pneumoniae* | ND |  | No growth |  | No agreement (PPID > MC) |
| 089 | CAP | Cardiovascular (CAD); Cardiovascular (Heart Failure); Cardiovascular (Hypertension) | *K. pneumoniae* | ND |  | No growth |  | No agreement (PPID > MC) |
| 090 | HCAP | Hepatic (Liver Cirrhosis) | *K. pneumoniae* | ND |  | No growth |  | No agreement (PPID > MC) |
| 091 | CAP | Cardiovascular (Hypertension); Endocrine (Diabetes) | *K. pneumoniae* | ND |  | No growth |  | No agreement (PPID > MC) |
| 092 | CAP | Cardiovascular (Hypertension) | *P. aeruginosa* | ND |  | Mixed flora |  | No agreement (PPID > MC) |
| 093 | CAP | No Comorbidity | *K. pneumoniae* | ND |  | Mixed flora |  | No agreement (PPID > MC) |
| 094 | CAP | Cardiovascular (Heart Failure); Endocrine (Diabetes); Respiratory (COPD) | *M. catarrhalis* | ND |  | Normal pharyngeal flora |  | No agreement (PPID > MC) |
| 095 | CAP | Cardiovascular (Heart Failure); Cardiovascular (CAD) | *E. coli* | ND |  | Normal pharyngeal flora |  | No agreement (PPID > MC) |
| 096 | CAP | Cancer; Cardiovascular (CAD); Cardiovascular (Hypertension) | *S. pneumoniae* | ND |  | Normal pharyngeal flora |  | No agreement (PPID > MC) |
| 097 | CAP | Cancer; Endocrine (Diabetes) | *E. coli* | ND |  | Normal pharyngeal flora |  | No agreement (PPID > MC) |
| 098 | CAP | Cancer; Cardiovascular (Hypertension); Endocrine (Diabetes) | *K. pneumoniae* | ND |  | Normal pharyngeal flora |  | No agreement (PPID > MC) |
| 099 | CAP | Cardiovascular (Hypertension); Endocrine (Diabetes); Respiratory (COPD); Hepatic (Liver Cirrhosis) | *L. pneumophila* | ND |  | No growth |  | Full agreement |
| 100 | CAP | Cardiovascular (CAD); Cardiovascular (Hypertension); Endocrine (Diabetes) | *K. pneumoniae, P. aeruginosa* | ND |  | No growth |  | No agreement (PPID > MC) |
| 101 | CAP | Cancer | *B. pertussis,*  *K. pneumoniae* | ND |  | No growth |  | No agreement (PPID > MC) |
| 102 | CAP | Cardiovascular (Heart Failure) | *K. pneumoniae, S. aureus* | ND |  | No growth |  | No agreement (PPID > MC) |
| 103 | HCAP | Cardiovascular (CAD); Cardiovascular (Heart Failure); Endocrine (Diabetes) | *K. pneumoniae, P. aeruginosa* | ND |  | No growth |  | No agreement (PPID > MC) |
| 104 | CAP | Cancer; Cardiovascular (CAD); Cardiovascular (Hypertension); Endocrine (Diabetes) | *H. influenzae,*  *K. pneumoniae* | ND |  | No growth |  | No agreement (PPID > MC) |
| 105 | HCAP | Cardiovascular (CAD); Cardiovascular (Heart Failure); Cardiovascular (Hypertension); Endocrine (Diabetes) | *E. coli,*  *K. pneumoniae* | ND |  | No growth |  | No agreement (PPID > MC) |
| 106 | CAP | Cancer | *E. coli,*  *K. pneumoniae* | ND |  | No growth |  | No agreement (PPID > MC) |
| 107 | CAP | Cancer; Cardiovascular (CAD); Cardiovascular (Hypertension) | *E. coli,*  *K. pneumoniae* | ND |  | No growth |  | No agreement (PPID > MC) |
| 108 | CAP | Cardiovascular (CAD); Cardiovascular (Hypertension); Respiratory (COPD) | *A. baumannii,*  *K. pneumoniae* | ND |  | No growth |  | No agreement (PPID > MC) |
| 109 | HCAP | Cancer; Respiratory (COPD) | *H. influenzae,*  *K. pneumoniae* | ND |  | Mixed flora |  | No agreement (PPID > MC) |
| 110 | CAP | Cardiovascular (CAD); Endocrine (Diabetes) | *H. influenzae,*  *S. pneumoniae* | ND |  | Mixed flora |  | No agreement (PPID > MC) |
| 111 | HCAP | Cardiovascular (Heart Failure); Cardiovascular (Hypertension) | *E. coli,*  *K. pneumoniae* | ND |  | Normal pharyngeal flora |  | No agreement (PPID > MC) |
| 112 | CAP | Endocrine (Diabetes) | *K. pneumoniae, P. aeruginosa* | ND |  | Normal pharyngeal flora |  | No agreement (PPID > MC) |
| 113 | CAP | No Comorbidity | *E. coli,*  *K. pneumoniae* | ND |  | Normal pharyngeal flora |  | No agreement (PPID > MC) |
| 114 | CAP | No Comorbidity | *H. influenzae,*  *K. pneumoniae, S. pneumoniae* | ND |  | No growth |  | No agreement (PPID > MC) |
| 115 | HAP | Cardiovascular (Heart Failure); Cardiovascular (Hypertension); Endocrine (Diabetes) | *E. coli,*  *K. pneumoniae, P. aeruginosa* | ND |  | No growth |  | No agreement (PPID > MC) |
| 116 | CAP | Cardiovascular (CAD) | *K. pneumoniae,*  *P. aeruginosa,*  *S. pneumoniae* | ND |  | No growth |  | No agreement (PPID > MC) |
| 117 | CAP | No Comorbidity | *H. influenzae,*  *K. pneumoniae, M. catarrhalis, S. aureus* | ND |  | No growth |  | No agreement (PPID > MC) |
| 118 | HCAP | Cardiovascular (CAD); Cardiovascular (Heart Failure); Cardiovascular (Hypertension); Endocrine (Diabetes) | *E. coli,*  *H. influenzae,*  *K. pneumoniae,*  *P. aeruginosa,*  *S. maltophilia* | ND |  | Mixed flora |  | No agreement (PPID > MC) |
| 119 | HAP | Cardiovascular (Hypertension); Endocrine (Diabetes) | *A. baumannii*  *E. coli,*  *K. pneumoniae,*  *P. aeruginosa,*  *S. maltophilia* | ND |  | Mixed flora |  | No agreement (PPID > MC) |
| 120 | CAP | Cardiovascular (Heart Failure) |  | InfA |  | No growth |  | Full agreement |
| 121 | CAP | Cardiovascular (Hypertension) |  | InfA |  | Mixed flora |  | Full agreement |
| 122 | CAP | No Comorbidity |  | InfA |  | Mixed flora |  | Full agreement |
| 123 | CAP | Cardiovascular (Hypertension); Endocrine (Diabetes); Cardiovascular (CAD) |  | InfA |  | Normal pharyngeal flora |  | Full agreement |
| 124 | CAP | No Comorbidity |  | SCV2 |  | No growth |  | Full agreement |
| 125 | CAP | No Comorbidity |  | SCV2 |  | No growth |  | Full agreement |
| 126 | CAP | No Comorbidity |  | SCV2 |  | No growth |  | Full agreement |
| 127 | CAP | No Comorbidity |  | SCV2 |  | Normal pharyngeal flora |  | Full agreement |
| 128 | CAP | No Comorbidity |  | SCV2 |  | Normal pharyngeal flora |  | Full agreement |
| 129 | CAP | No Comorbidity |  | SCV2 |  | Normal pharyngeal flora |  | Full agreement |
| 130 | CAP | No Comorbidity |  | SCV2 |  | Normal pharyngeal flora |  | Full agreement |
| 131 | CAP | No Comorbidity |  | SCV2 |  | Normal pharyngeal flora |  | Full agreement |
| 132 | CAP | No Comorbidity |  | SCV2 |  | Normal pharyngeal flora |  | Full agreement |
| 133 | CAP | Cardiovascular (Hypertension); Renal disease | *H. influenzae,*  *K. pneumoniae* | InfA |  | No growth |  | No agreement (PPID > MC) |
| 134 | CAP | Endocrine (Diabetes) | *S. aureus* | InfA |  | Mixed flora |  | No agreement (PPID > MC) |
| 135 | CAP | No Comorbidity | *K. pneumoniae, P. aeruginosa* | SCV2 |  | No growth |  | No agreement (PPID > MC) |

ND, not detected; InfA, influenza A virus; HPIV-3, human parainfluenza virus type 3; RSV-B, respiratory syncytial virus type B; Rhino, rhinovirus; SCV2, SARS-CoV-2.

*^a^* Non-PPID targets or pathogens not routinely cultured (viruses, atypical or fastidious bacteria) are underlined.

*^b‍‍^*Pathogens detected by both methods are shown in bold.

*^c‍‍^*Agreements are categorized solely within the scope of the targets common to both the PPID and the MC methods and defined as follows:

Full agreement: PPID and MC agree on all

Partial agreement (PPID > MC): At least one target is detected by both, and PPID detects all MC-positive and more.

Partial agreement (PPID ≸ MC): At least one target is detected by both, and each detects at least one not detected by the other.

Partial agreement (MC > PPID): At least one target is detected by both, and MC detects all PPID-positive and more.

**Table S6 (Continued).** Pathogen identification in clinical specimens using the PPID assay and MC methods (N = 135)

No agreement (PPID > MC): At least one target is detected by PPID and none by MC.

No agreement (PPID ≸ MC): Each method detects at least one target, and none is detected by both.

No agreement (MC > PPID): At least one target is detected by MC and none by PPID.

*^d‍‍^*Pathogens detected only by the PPID assay were analyzed by singleplex qPCR.

**Table S7.** Distribution of pathogens in single and multiple infections identified by the PPID assay

|  |  |  |  |  |  |
| --- | --- | --- | --- | --- | --- |
| **Pathogen** | **Pathogen detection by PPID, N (%)** | | | | |
|  | **Overall** | **Single pathogen** | | **Multiple pathogens** | |
| Positive | 105 | 54 |  | 51 |  |
| *K. pneumoniae* | 57 | 17 | (31.5)‍*^a^* | 40 | (78.4)‍*^b^* |
| *P. aeruginosa* | 24 | 3 | (5.6)‍*^a^* | 21 | (41.2)‍*^b^* |
| *E. coli* | 22 | 4 | (7.4)‍*^a^* | 18 | (35.3)‍*^b^* |
| *H. influenzae* | 19 | 6 | (11.1)‍*^a^* | 13 | (25.5)‍*^b^* |
| *A. baumannii* | 15 | 3 | (5.6)‍*^a^* | 12 | (23.5)‍*^b^* |
| *S. aureus* | 9 | 0 |  | 9 | (17.6)‍*^b^* |
| *S. maltophilia* | 7 | 0 |  | 7 | (13.7)‍*^b^* |
| *S. pneumoniae* | 7 | 2 | (3.7)‍*^a^* | 5 | (9.8)‍*^b^* |
| *M. catarrhalis* | 5 | 2 | (3.7)‍*^a^* | 3 | (5.9)‍*^b^* |
| *B. pertussis* | 1 | 0 |  | 1 | (2.0)‍*^b^* |
| *L. pneumophila* | 1 | 1 | (1.9)‍*^a^* | 0 |  |
| SARS-CoV-2 | 10 | 9 | (16.7‍)*^a^* | 1 | (2.0)‍*^b^* |
| Influenza A virus | 9 | 5 | (9.3)‍‍*^a^* | 4 | (7.8)‍*^b^* |
| Rhinovirus | 1 | 1 | (1.9)‍*^a^* | 0 |  |
| Respiratory syncytial virus type B | 1 | 1 | (1.9)‍*^a^* | 0 |  |
| Human parainfluenza virus type 3 | 1 | 0 |  | 1 | (2.0)‍*^b^* |

**Table S7 (Continued).** Distribution of pathogens in single and multiple infections identified by the PPID assay

*^a‍‍^*The percentages represent the detection rates of specific pathogens in single infections, using the total number of single-pathogen cases (N=54) as the denominator.

*^b‍‍^*The percentages represent the detection rates of specific pathogens in polymicrobial infections, using the total number of multiple-pathogen cases (N=51) as the denominator.

**Table S8.** Identification of target pathogens common between the PPID assay and MC methods

| **Spec­­**­**i­men** | ***A. bau­mannii*** | ***E. coli*** | ***H. in­flu­en­za­e*** | ***H. in­flu­en*** ***­zae type b*** | ***K. pneu­mo­ni­a­e*** | ***M. ca­tar-rha­lis*** | ***N. me­nin­gi­ti­dis*** | ***P. a­e­ru­gi­no­sa*** | ***S. aureus*** | ***S. mal­to­phi­li­a*** | ***S. pneu­mo­ni­a­e*** | ***S. py­o­ge­nes*** | **Agreement Category** |
| --- | --- | --- | --- | --- | --- | --- | --- | --- | --- | --- | --- | --- | --- |
| 001 |  |  |  |  |  |  |  |  |  |  |  |  | Full agreement |
| 002 |  |  |  |  |  |  |  |  |  |  |  |  | Full agreement |
| 003 |  |  |  |  |  |  |  |  |  |  |  |  | Full agreement |
| 004 |  |  |  |  |  |  |  |  |  |  |  |  | Full agreement |
| 005 |  |  |  |  |  |  |  |  |  |  |  |  | Full agreement |
| 006 |  |  |  |  |  |  |  |  |  |  |  |  | Full agreement |
| 007 |  |  |  |  |  |  |  |  |  |  |  |  | Full agreement |
| 008 |  |  |  |  |  |  |  |  |  |  |  |  | Full agreement |
| 009 |  |  |  |  |  |  |  |  |  |  |  |  | Full agreement |
| 010 |  |  |  |  |  |  |  |  |  |  |  |  | Full agreement |
| 011 |  |  |  |  |  |  |  |  |  |  |  |  | Full agreement |
| 012 |  |  |  |  |  |  |  |  |  |  |  |  | Full agreement |
| 013 |  |  |  |  |  |  |  |  |  |  |  |  | Full agreement |
| 014 |  |  |  |  |  |  |  |  |  |  |  |  | Full agreement |
| 015 |  |  |  |  |  |  |  |  |  |  |  |  | Full agreement |
| 016 |  |  |  |  |  |  |  |  |  |  |  |  | Full agreement |
| 017 |  |  |  |  |  |  |  |  |  |  |  |  | Full agreement |
| 018 |  |  |  |  |  |  |  |  |  |  |  |  | Full agreement |
| 019 |  |  |  |  |  |  |  |  |  |  |  |  | Full agreement |
| 020 | BOTH |  |  |  |  |  |  |  |  |  |  |  | Full agreement |
| 021 |  |  |  |  | BOTH |  |  |  |  |  |  |  | Full agreement |
| 022 |  |  |  |  | BOTH |  |  |  |  |  |  |  | Full agreement |
| 023 |  |  |  |  |  | BOTH |  |  |  |  |  |  | Full agreement |
| 024 |  |  |  |  | BOTH |  |  |  |  |  |  |  | Full agreement |
| 025 |  |  | BOTH |  |  |  |  |  |  |  |  |  | Full agreement |
| 026 |  |  | BOTH |  |  |  |  |  |  |  |  |  | Full agreement |
| 027 |  |  |  |  | BOTH |  |  |  |  |  |  |  | Full agreement |
| 028 |  |  |  |  |  |  |  |  |  |  | BOTH |  | Full agreement |
| 029 |  |  |  |  | BOTH |  |  |  |  |  |  |  | Full agreement |
| 030 |  |  |  |  | BOTH |  |  |  |  |  |  |  | Full agreement |
| 031 |  |  |  |  | BOTH |  |  |  |  |  |  |  | Full agreement |
| 032 |  | BOTH |  |  |  |  |  |  |  |  |  |  | Full agreement |
| 033 |  |  |  |  |  |  |  | BOTH |  |  |  |  | Full agreement |
| 034 |  |  |  |  |  |  |  | BOTH |  |  |  |  | Full agreement |
| 035 |  | PPID |  |  | BOTH |  |  |  |  |  |  |  | Partial agreement (PPID > MC) |
| 036 |  |  |  |  | BOTH | PPID |  |  |  |  |  |  | Partial agreement (PPID > MC) |
| 037 |  |  | PPID |  | BOTH |  |  |  |  |  |  |  | Partial agreement (PPID > MC) |
| 038 |  | PPID |  |  | BOTH | PPID |  |  |  |  |  |  | Partial agreement (PPID > MC) |
| 039 |  | BOTH |  |  | PPID |  |  | PPID |  |  |  |  | Partial agreement (PPID > MC) |
| 040 |  |  | PPID |  |  |  |  | PPID |  | BOTH |  |  | Partial agreement (PPID > MC) |
| 041 |  | PPID |  |  | PPID |  |  |  | BOTH |  |  |  | Partial agreement (PPID > MC) |
| 042 | PPID |  | PPID |  | BOTH |  |  |  |  |  |  |  | Partial agreement (PPID > MC) |
| 043 | PPID |  |  |  |  |  |  | PPID |  | BOTH |  |  | Partial agreement (PPID > MC) |
| 044 | BOTH |  |  |  |  |  |  |  | PPID | PPID |  |  | Partial agreement (PPID > MC) |
| 045 | BOTH | PPID |  |  | PPID |  |  |  | PPID |  |  |  | Partial agreement (PPID > MC) |
| 046 | PPID |  |  |  | BOTH |  |  | PPID |  |  | PPID |  | Partial agreement (PPID > MC) |
| 047 |  |  |  |  | PPID |  |  | BOTH |  | BOTH |  |  | Partial agreement (PPID > MC) |
| 048 |  | PPID |  |  | BOTH |  |  | PPID | BOTH |  |  |  | Partial agreement (PPID > MC) |
| 049 |  |  |  |  |  |  |  |  | BOTH |  |  |  | Full agreement |
| 050 |  |  | PPID |  | BOTH |  |  |  |  |  |  |  | Partial agreement (PPID > MC) |
| 051 | PPID |  |  |  | PPID |  |  | BOTH |  |  |  |  | Partial agreement (PPID > MC) |
| 052 | BOTH |  |  |  |  |  |  |  | MC |  |  |  | Partial agreement (MC > PPID) |
| 053 | MC |  |  |  |  |  |  | BOTH |  |  | PPID |  | Partial agreement (PPID ≸ MC) |
| 054 |  | BOTH |  |  |  |  |  | PPID |  | MC |  |  | Partial agreement (PPID ≸ MC) |
| 055 | MC | PPID |  |  | PPID |  |  |  |  |  |  |  | No agreement (PPID ≸ MC) |
| 056 | MC | PPID |  |  |  |  |  |  |  |  |  |  | No agreement (PPID ≸ MC) |
| 057 | PPID |  |  |  |  |  |  | MC |  |  |  |  | No agreement (PPID ≸ MC) |
| 058 | PPID |  |  |  |  |  |  | PPID |  | MC |  |  | No agreement (PPID ≸ MC) |
| 059 | PPID |  |  |  | PPID |  |  | PPID | MC |  |  |  | No agreement (PPID ≸ MC) |
| 060 | PPID |  |  |  | PPID |  |  |  |  | MC |  |  | No agreement (PPID ≸ MC) |
| 061 |  |  |  |  | PPID |  |  |  |  |  |  |  | No agreement (PPID > MC) |
| 062 |  |  |  |  | PPID |  |  |  |  | PPID |  |  | No agreement (PPID > MC) |
| 063 |  | PPID |  |  | PPID |  |  |  |  |  |  |  | No agreement (PPID > MC) |
| 064 |  |  |  |  | PPID |  |  |  | PPID |  |  |  | No agreement (PPID > MC) |
| 065 |  |  | PPID |  |  |  |  | PPID |  |  |  |  | No agreement (PPID > MC) |
| 066 | PPID | PPID | PPID |  |  |  |  | PPID |  |  |  |  | No agreement (PPID > MC) |
| 067 |  |  |  |  |  |  |  |  |  |  |  |  | Full agreement |
| 068 |  |  |  |  |  |  |  |  |  |  |  |  | Full agreement |
| 069 |  |  |  |  |  |  |  | MC |  |  |  |  | No agreement (MC > PPID) |
| 070 |  |  |  |  |  |  |  |  | MC |  |  |  | No agreement (MC > PPID) |
| 071 |  |  | MC |  |  |  |  |  |  |  |  |  | No agreement (MC > PPID) |
| 072 | MC |  |  |  |  |  |  |  |  |  |  |  | No agreement (MC > PPID) |
| 073 |  |  |  |  |  | MC |  |  |  |  |  |  | No agreement (MC > PPID) |
| 074 |  |  |  |  |  |  |  |  |  | MC |  |  | No agreement (MC > PPID) |
| 075 |  |  | MC |  |  |  |  |  |  |  |  |  | No agreement (MC > PPID) |
| 076 |  |  |  |  |  |  |  |  |  |  |  |  | Full agreement |
| 077 |  |  |  |  |  |  |  |  |  |  |  |  | Full agreement |
| 078 |  |  |  |  |  |  |  |  |  |  |  |  | Full agreement |
| 079 |  |  |  |  |  |  |  |  |  |  |  |  | Full agreement |
| 080 |  |  |  |  |  |  |  |  |  |  |  |  | Full agreement |
| 081 |  |  | PPID |  |  |  |  |  |  |  |  |  | No agreement (PPID > MC) |
| 082 |  |  | PPID |  |  |  |  |  |  |  |  |  | No agreement (PPID > MC) |
| 083 |  |  | PPID |  |  |  |  |  |  |  |  |  | No agreement (PPID > MC) |
| 084 |  |  | PPID |  |  |  |  |  |  |  |  |  | No agreement (PPID > MC) |
| 085 |  |  |  |  | PPID |  |  |  |  |  |  |  | No agreement (PPID > MC) |
| 086 |  |  |  |  | PPID |  |  |  |  |  |  |  | No agreement (PPID > MC) |
| 087 |  |  |  |  | PPID |  |  |  |  |  |  |  | No agreement (PPID > MC) |
| 088 |  |  |  |  | PPID |  |  |  |  |  |  |  | No agreement (PPID > MC) |
| 089 |  |  |  |  | PPID |  |  |  |  |  |  |  | No agreement (PPID > MC) |
| 090 |  |  |  |  | PPID |  |  |  |  |  |  |  | No agreement (PPID > MC) |
| 091 |  |  |  |  | PPID |  |  |  |  |  |  |  | No agreement (PPID > MC) |
| 092 |  |  |  |  |  |  |  | PPID |  |  |  |  | No agreement (PPID > MC) |
| 093 |  |  |  |  | PPID |  |  |  |  |  |  |  | No agreement (PPID > MC) |
| 094 |  |  |  |  |  | PPID |  |  |  |  |  |  | No agreement (PPID > MC) |
| 095 |  | PPID |  |  |  |  |  |  |  |  |  |  | No agreement (PPID > MC) |
| 096 |  |  |  |  |  |  |  |  |  |  | PPID |  | No agreement (PPID > MC) |
| 097 |  | PPID |  |  |  |  |  |  |  |  |  |  | No agreement (PPID > MC) |
| 098 |  |  |  |  | PPID |  |  |  |  |  |  |  | No agreement (PPID > MC) |
| 099 |  |  |  |  |  |  |  |  |  |  |  |  | Full agreement |
| 100 |  |  |  |  | PPID |  |  | PPID |  |  |  |  | No agreement (PPID > MC) |
| 101 |  |  |  |  | PPID |  |  |  |  |  |  |  | No agreement (PPID > MC) |
| 102 |  |  |  |  | PPID |  |  |  | PPID |  |  |  | No agreement (PPID > MC) |
| 103 |  |  |  |  | PPID |  |  | PPID |  |  |  |  | No agreement (PPID > MC) |
| 104 |  |  | PPID |  | PPID |  |  |  |  |  |  |  | No agreement (PPID > MC) |
| 105 |  | PPID |  |  | PPID |  |  |  |  |  |  |  | No agreement (PPID > MC) |
| 106 |  | PPID |  |  | PPID |  |  |  |  |  |  |  | No agreement (PPID > MC) |
| 107 |  | PPID |  |  | PPID |  |  |  |  |  |  |  | No agreement (PPID > MC) |
| 108 | PPID |  |  |  | PPID |  |  |  |  |  |  |  | No agreement (PPID > MC) |
| 109 |  |  | PPID |  | PPID |  |  |  |  |  |  |  | No agreement (PPID > MC) |
| 110 |  |  | PPID |  |  |  |  |  |  |  | PPID |  | No agreement (PPID > MC) |
| 111 |  | PPID |  |  | PPID |  |  |  |  |  |  |  | No agreement (PPID > MC) |
| 112 |  |  |  |  | PPID |  |  | PPID |  |  |  |  | No agreement (PPID > MC) |
| 113 |  | PPID |  |  | PPID |  |  |  |  |  |  |  | No agreement (PPID > MC) |
| 114 |  |  | PPID |  | PPID |  |  |  |  |  | PPID |  | No agreement (PPID > MC) |
| 115 |  | PPID |  |  | PPID |  |  | PPID |  |  |  |  | No agreement (PPID > MC) |
| 116 |  |  |  |  | PPID |  |  | PPID |  |  | PPID |  | No agreement (PPID > MC) |
| 117 |  |  | PPID |  | PPID | PPID |  |  | PPID |  |  |  | No agreement (PPID > MC) |
| 118 |  | PPID | PPID |  | PPID |  |  | PPID |  | PPID |  |  | No agreement (PPID > MC) |
| 119 | PPID | PPID |  |  | PPID |  |  | PPID |  | PPID |  |  | No agreement (PPID > MC) |
| 120 |  |  |  |  |  |  |  |  |  |  |  |  | Full agreement |
| 121 |  |  |  |  |  |  |  |  |  |  |  |  | Full agreement |
| 122 |  |  |  |  |  |  |  |  |  |  |  |  | Full agreement |
| 123 |  |  |  |  |  |  |  |  |  |  |  |  | Full agreement |
| 124 |  |  |  |  |  |  |  |  |  |  |  |  | Full agreement |
| 125 |  |  |  |  |  |  |  |  |  |  |  |  | Full agreement |
| 126 |  |  |  |  |  |  |  |  |  |  |  |  | Full agreement |
| 127 |  |  |  |  |  |  |  |  |  |  |  |  | Full agreement |
| 128 |  |  |  |  |  |  |  |  |  |  |  |  | Full agreement |
| 129 |  |  |  |  |  |  |  |  |  |  |  |  | Full agreement |
| 130 |  |  |  |  |  |  |  |  |  |  |  |  | Full agreement |
| 131 |  |  |  |  |  |  |  |  |  |  |  |  | Full agreement |
| 132 |  |  |  |  |  |  |  |  |  |  |  |  | Full agreement |
| 133 |  |  | PPID |  | PPID |  |  |  |  |  |  |  | No agreement (PPID > MC) |
| 134 |  |  |  |  |  |  |  |  | PPID |  |  |  | No agreement (PPID > MC) |
| 135 |  |  |  |  | PPID |  |  | PPID |  |  |  |  | No agreement (PPID > MC) |

**Table S8 (Continued).** Identification of target pathogens common between the PPID assay and MC methods

PPID, the pathogen is detected by the PPID assay; MC, the pathogen is detected by the MC method; BOTH, the pathogen is detected by both methods

**Table S9.** Pathogens detected only by the PPID assay

| **Pathogen** | **Detection by PPID** | |
| --- | --- | --- |
|  | **Number, N** | **Percentage, %*^a^*** |
| SCV2 | 10 | 7.4 |
| InfA | 9 | 6.7 |
| *B. pertussis* | 1 | 0.7 |
| HPIV-3 | 1 | 0.7 |
| *L. pneumophila* | 1 | 0.7 |
| Rhino | 1 | 0.7 |
| RSV-B | 1 | 0.7 |
| *C. pneumoniae* | 0 | 0.0 |
| HPIV-1 | 0 | 0.0 |
| HPIV-2 | 0 | 0.0 |
| HPIV-4 | 0 | 0.0 |
| InfB | 0 | 0.0 |
| *M. pneumoniae* | 0 | 0.0 |
| *M. tuberculosis* | 0 | 0.0 |
| RSV-A | 0 | 0.0 |

*^a‍‍^*The percentages are calculated using the total of 135 ETA specimens as the denominator.

**Table S10.** Pathogens detected only by the MC methods

| **Pathogen** | **Detection by MC** | |
| --- | --- | --- |
|  | **Number, N** | **Percentage, %*^a^*** |
| *C. albicans* | 14 | 10.4 |
| Non- *C. albicans* yeasts | 5 | 3.7 |
| *S. agalactiae* | 3 | 2.2 |
| *S. marcescens* | 3 | 2.2 |
| Beta-Streptococcus Non-A,B,D. | 2 | 1.5 |
| *Cupriavidus pauculus* | 1 | 0.7 |
| *E. anophelis* | 1 | 0.7 |
| *E. cloacae* | 1 | 0.7 |
| *Elizabethkingia anophelis* | 1 | 0.7 |
| *Elizabethkingia meningoseptica* | 1 | 0.7 |
| *Enterobacter cloacae* | 1 | 0.7 |
| *Klebsiella oxytoca* | 1 | 0.7 |
| *Morganella morganii* | 1 | 0.7 |
| Non tuberculosis Mycobacterium | 1 | 0.7 |
| *P. stuartii* | 1 | 0.7 |
| *Proteus mirabilis* | 1 | 0.7 |
| *Providencia stuartii* | 1 | 0.7 |
| *Streptococcus agalactiae* | 1 | 0.7 |

*^a‍‍^*The percentages are calculated using the total of 135 ETA specimens as the denominator.

**Table S11.** Primers and probes used in the single plex confirmation qPCR

| **Respiratory pathogen** | **Probe set** | **Target gene** | **Primers & Probes‍‍^a^** | **Sequence‍‍^b^ (5' to 3')** |
| --- | --- | --- | --- | --- |
| *Acinetobacter baumannii* | Aba | *oxa-51* | Aba-oxa51-F | CTCGTGCTTCGACCGAGTAT |
|  |  |  | Aba-oxa51-R | AACCAACACGCTTCACTTCC |
|  |  |  | Aba-oxa51-P | ATGAAAGCTTCCGCTATTCCGGTT |
| *Bordetella pertussis* | Bpe | *transposase* | Bpe-IS481-F | GGCATCGACCCCACCAA |
|  |  |  | Bpe-IS481-R | GGTTGTATTCGTCCAGGTTGAGT |
|  |  |  | Bpe-IS481-P | TCGGGCGCGCTGTACCCATCT |
| *E. coli* | Eco | *uidA* | Eco-uidA-F | CGGCGTGGTGTAGAGCATT |
|  |  |  | Eco-uidA -R | GCAGTCTTACTTCCATGATTTCTTTAAC |
|  |  |  | Eco-uidA -P | CGCTGCGATGGATCCCGG |
| *Haemophilus influenzae* Type A | Hin | *hpd* | Hinf-hpd-F | GGTTAAATATGCCGATGGTGTTG |
|  |  |  | Hinf-hpd-R | TGCATCTTTACGCACGGTGTA |
|  |  |  | Hinf-hpd-P | SUN-TTGTGTACACTCCGTTGGTAAAAGAACTTGCAC |
| *Klebsiella pneumoniae* | Kpn | *khe (Hemolycin)* | Kpn-khe-F | GATGAAACGACCTGATTGCATTC |
|  |  |  | Kpn-khe-R | CCGGGCTGTCGGGATAAG |
|  |  |  | Kpn-khe-P | CGCGAACTGGAAGGGCCCG |
| *Legionella pneumophila* | Lpn | *mip* | Lpn-mip-F | ATGAAGATGAAATTGGTGACTGC |
|  |  |  | Lpn-mip-R | TTCCCAAATCGGCACCAATG |
| *Moraxella catarrhalis* | Mca | *copB* | Mca-copB-F | CGTGTTGACCGTTTTGACTTT |
|  |  |  | Mca-copB-R | TAGATTAGGTTACCGCTGACG |
|  |  |  | Mca-copB-P | ACCGACATCAACCCAAGCTTTGG |
| *Pseudomonas aeruginosa* | Pae | *23S rDNA* | Pae-23S-F | TCCAAGTTTAAGGTGGTAGGCTG |
|  |  |  | Pae-23S-R | ACCACTTCGTCATCTAAAAGACGAC |
|  |  |  | Pae-23S-P | AGGTAAATCCGGGGTTTCAAGGCC |
| *Staphylococcus aureus* | Sau | *16S rDNA* | Sau-16S-F | TGGCGGCGTGCCTAATA |
|  |  |  | Sau-16S-R | CGCTAACATCAGAGAAGCAAGCT |
|  |  |  | Sau-16S-P | ATGCAAGTCGAGCGAACGGACGAG |
| *Stenotrophomonas maltophilia* | Sma | *fdnG* | Sma-fdnG-F | AAGGACAAGGCGATGACCATC |
|  |  |  | Sma-fdnG-R2 | CCCCACCACGACTTCATCA |
|  |  |  | Sma-fdnG-P | CAGAACGACATCTGGTTGGCG |
| *Streptococcus pneumoniae* | Spn | *lytA* | Spn lytA-F | AAGGTGCCATGAAGACAGG |
|  |  |  | Spn lytA-R | TTTGATACCATGGCGCCTTCTTT |
|  |  |  | Spn lytA-P | TGTGTCCTTGTACTTGACCCAGC |
| *Streptococcus pyogenes* | Spy | *GAS* | Spy-GAS-F | TGGATGTGGTTGCAGGTTTAGAC |
|  |  |  | Spy-GAS-R | CGGGCAAGTAGTTCTTCAATGG |
|  |  |  | Spy-GAS-P | CGGTGCAGACGACTATATTGTTAAACC |
| Influenza A virus | InfA | *M1* | FluA-M1-F | AAGACCAATCCTGTCACCTCTGA |
|  |  |  | FluA-M1-R | CAAAGCGTCTACGCTGCAGTCC |
|  |  |  | FluA-M1-P | CACCGTGCCCAGTGAGCGA |
| Rhinovirus | Rhino | *5'UTR* | Rhino-5UTR-F | TGGACAGGGTGTGAAGAGC |
|  |  |  | Rhino-5UTR-R | CAAAGTAGTCGGTCCCATCC |
|  |  |  | Rhino-5UTR-P | TCCTCCGGCCCCTGAATG |
| Respiratory Syncytial virus Type B | RSVB | *N* | RSVB-N-F | AAGATGCAAATCATAAATTCACAGGA |
|  |  |  | RSVB-N-R | TGATATCCAGCATCTTTAAGTATCTTTATAGTG |
|  |  |  | RSVB-N-P | AGGTATGTTATATGCTATGTCCAGGTTAGGAAGGGAA |
| Human parainfluenza virus Type 3 | HPIV3 | *HN* | HPIV3-HN-F | GTCTGGTCTTCCATCTTTGATG |
|  |  |  | HPIV3-HN-R | CCTATATCCTGGCAACCTCG |
|  |  |  | HPIV3-HN-P | TGCCAACGACTGTTGATGGC |
| SARS-CoV-2 | SCV2 | *nsp2* | SCV2-nsp2-F | ATGCATTTGCATCAGAGGCT |
|  |  |  | SCV2-nsp2-R | TTGTTATAGCGGCCTTCTGT |
|  |  |  | SCV2-nsp2-P | TCTCCCGCACTCTTGAAACT |

*Oxa-51* (class D β-lactamase gene), *khe* (hemolysin gene), *23S rDNA* (23S ribosomal DNA), *16S rDNA* (16S ribosomal DNA), *GAS* (group A Streptococcus–specific gene), and *nsp2* (nonstructural protein 2 gene).

**Table S12.** Clinical accuracy of the PPID assay, using the MC methods as the comparator methods

| Respiratory bacteria (PPID-targets) | PPID  positive | MC  positive | Compare PPID to MC | | | | | | |
| --- | --- | --- | --- | --- | --- | --- | --- | --- | --- |
|  |  |  | PPID+MC+ | PPID−MC− | PPID+MC− | PPID−MC+ | PPA  (95% CI) | NPA  (95% CI) | OPA  (95% CI) |
| *Klebsiella pneumoniae* | 57 | 15 | 15 | 78 | 42 | 0 | 100.0%  (79.6%, 100.0%) | 65.0%  (56.1%, 72.9%) | 68.9%  (60.6%, 76.1%) |
| *Pseudomonas aeruginosa* | 24 | 7 | 5 | 109 | 19 | 2 | 71.4%  (35.9%, 91.8%) | 85.2%  (78.0%, 90.3%) | 84.4%  (77.4%, 89.6%) |
| *Escherichia coli, E. coli* | 22 | 3 | 3 | 113 | 19 | 0 | 100.0%  (43.9%, 100.0%) | 85.6%  (78.6%, 90.6%) | 85.9%  (79.1%, 90.8%) |
| *Haemophilus influenzae* | 19 | 4 | 2 | 114 | 17 | 2 | 50.0%  (15.0%, 85.0%) | 87.0%  ((80.2%, 91.7%) | 85.9%  (79.1%, 90.8%) |
| *Acinetobacter baumannii* | 15 | 8 | 4 | 116 | 11 | 4 | 50.0%  (21.5%, 78.5%) | 91.3%  (85.2%, 95.1%) | 88.9%  (82.5%, 93.2%) |
| *Staphylococcus aureus* | 9 | 6 | 3 | 123 | 6 | 3 | 50.0%  (18.8%, 81.2%) | 95.3%  (90.2%, 97.9%) | 93.3%  (87.8%, 96.5%) |
| *Stenotrophomonas maltophilia* | 7 | 7 | 3 | 124 | 4 | 4 | 42.9%  (15.8%, 75.0%) | 96.9%  (92.2%, 98.8%) | 94.1%  (88.7%, 97.0%) |
| *Streptococcus pneumoniae* | 7 | 1 | 1 | 128 | 6 | 0 | 100.0%  (20.7%, 100.0%) | 95.5%  90.6%, 97.9%) | 95.6%  (90.6%, 97.9%) |
| *Moraxella catarrhalis* | 5 | 2 | 1 | 129 | 4 | 1 | 50.0%  (9.4%, 90.6%) | 97.0%  (92.5%, 98.8%) | 96.3%  (91.6%, 98.4%) |
| *Haemophilus influenzae* type b | 0 | 0 | 0 | 135 | 0 | 0 | NA | 100.0%  (97.2%, 100.0%) | 100.0%  (97.2%, 100.0%) |
| *Neisseria meningitidis* | 0 | 0 | 0 | 135 | 0 | 0 | NA | 100.0%  (97.2%, 100.0%) | 100.0%  (97.2%, 100.0%) |
| *Streptococcus pyogenes* | 0 | 0 | 0 | 135 | 0 | 0 | NA | 100.0%  (97.2%, 100.0%) | 100.0%  (97.2%, 100.0%) |

PPA, positive percent agreement; NPA, negative percent agreement; OPA, overall percent agreement.

NA, not applicable.
